# Mammalian transcriptional hotspots are enriched for tissue specific enhancers near cell type specific highly expressed genes and are predicted to act as transcriptional activator hubs

**DOI:** 10.1186/s12859-014-0412-0

**Published:** 2014-12-30

**Authors:** Anagha Joshi

**Affiliations:** Division of Developmental Biology, The Roslin Institute and Royal (Dick) School of Veterinary Studies, University of Edinburgh, Easter Bush Campus, Midlothian, EH25 8GR UK

**Keywords:** ChIP sequencing, Transcription hotspots, Transcription regulation, Data analysis

## Abstract

**Background:**

Transcriptional hotspots are defined as genomic regions bound by multiple factors. They have been identified recently as cell type specific enhancers regulating developmentally essential genes in many species such as worm, fly and humans. The in-depth analysis of hotspots across multiple cell types in same species still remains to be explored and can bring new biological insights.

**Results:**

We therefore collected 108 transcription-related factor (TF) ChIP sequencing data sets in ten murine cell types and classified the peaks in each cell type in three groups according to binding occupancy as singletons (low-occupancy), combinatorials (mid-occupancy) and hotspots (high-occupancy). The peaks in the three groups clustered largely according to the occupancy, suggesting priming of genomic loci for mid occupancy irrespective of cell type. We then characterized hotspots for diverse structural functional properties. The genes neighbouring hotspots had a small overlap with hotspot genes in other cell types and were highly enriched for cell type specific function. Hotspots were enriched for sequence motifs of key TFs in that cell type and more than 90% of hotspots were occupied by pioneering factors. Though we did not find any sequence signature in the three groups, the H3K4me1 binding profile had bimodal peaks at hotspots, distinguishing hotspots from mono-modal H3K4me1 singletons. In ES cells, differentially expressed genes after perturbation of activators were enriched for hotspot genes suggesting hotspots primarily act as transcriptional activator hubs. Finally, we proposed that ES hotspots might be under control of SetDB1 and not DNMT for silencing.

**Conclusion:**

Transcriptional hotspots are enriched for tissue specific enhancers near cell type specific highly expressed genes. In ES cells, they are predicted to act as transcriptional activator hubs and might be under SetDB1 control for silencing.

**Electronic supplementary material:**

The online version of this article (doi:10.1186/s12859-014-0412-0) contains supplementary material, which is available to authorized users.

## Background

Transcriptional control of gene expression via localisation and binding of transcription factors at the vicinity of gene loci lies at the heart of metazoan development. With the advances in sequencing protocols, ChIP sequencing is rapidly becoming the preferred tool to find genome-wide binding patterns (peaks) of a transcription factor in a given cell type. As ChIP sequencing protocols mature, peaks of multiple transcription factors have been characterized in single cell types to study combinatorial control [1]. One of the striking observations of these multi-TF studies was detection of so-called ‘transcriptional hotspots’. In 2006, Moorman *et al.* [2] generated genome-wide binding profiles of seven transcription factors in *D. melanogaster* and identified a subset of peaks bound by all seven TFs (hotspots). Of these 108 hotspots, when tested using transgenic assays, 94% acted as enhancers strongly activating the neighbouring developmentally important genes [3]. The presence of hotspot regions was further confirmed in other species such as worm [4] and humans [5]. Hotspots in *C. elegans* and *D. melanogaster* were enriched for sequence binding motifs of many TFs including signature motifs such as GAGA and Zelda [3]. However, analysis of human ENCODE data did not support these observations. In humans, hotspots were specifically deprived of cis-regulatory motifs and no signature motif similar to the ‘GAGA’ motif was found [5]. At the other end of the spectrum, most TF binding events were ‘singletons’ (bound by only one TF) accounting for nearly one third of the binding events in a cell type. These regions when studied in *D. melanogaster* transgenic assays did not drive patterned reporter gene expression leading to the conclusion that they do not act as strong developmental enhancers [6]. This leads to a series of questions about properties of both hotspots and singletons. For example, are they truly distinct genomic regions, do they have a characteristic sequence or chromatin signature?

In order to answer the above questions, we collected genome-wide binding patterns of multiple transcription-related factors in ten murine cell types. For each cell type, the peaks were classified into three groups: singleton genomic regions occupied by only one TF (low-occupancy), hotspot genomic regions occupied by most TFs under study (high-occupancy) and combinatorial genomic regions occupied by a combination of TFs (mid-occupancy). The genomic regions largely clustered according to the group suggesting distinct genomic regions marked for occupancy independent of cell type. The singletons and combinatorials were bound neighbouring similar genes in all cell types while hotspot peaks occurred near a distinct set of genes in each cell type and showed functional enrichment for cell type specific genes. Though hotspots were enriched for many TF sequence motifs, no signature motif such as GAGA motif was found in murine cell types. We identified H3K4me1 chromatin modification distinguishing hotspots from singletons where hotspots showed a bimodal H3K4me1 peak whereas singletons were mono-modal. Finally, we collected the differentially regulated genes after perturbations of multiple transcription-related factors in ES cells, to show that hotspots were preferentially bound by activators and not repressors. As genes differentially expressed after *Setdb1* knockout but not *Dnmt* knockout were enriched for ES hotspot genes we suggest that Setdb1 might be involved in silencing hotspots.

## Results and discussion

### Combinatorial binding events overlap across multiple cell types

**Figure 1:**
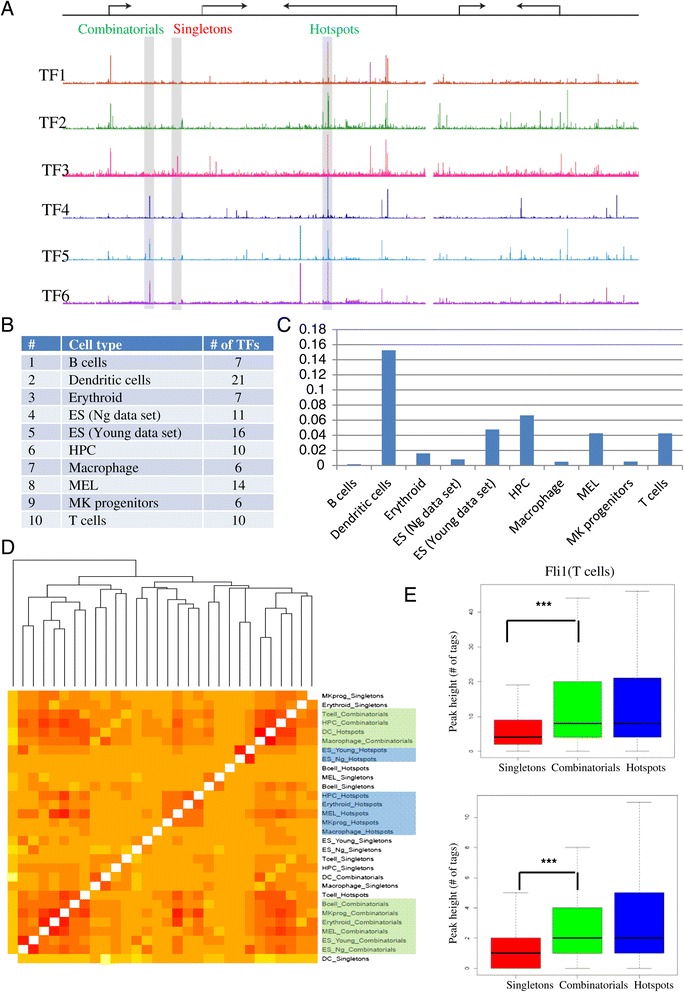
**Combinatorial binding events overlap across multiple cell types. A**. schematic diagram of a genomic region with genome tracks of six ChIP sequencing samples in a cell type marking singletons (bound by only one transcription-related factor), combinatorials (bound by few transcription-related factors) and hotspots (peaks bound by more than five transcription-related factors). **B**. A table listing all 10 cell types used along with the number of transcription factors **C**. bar graph of fraction of peaks in hotspots across 10 cell types. **D**. Heatmap of Pearson’s correlation coefficients of peaks in 30 sets (three groups of 10 cell types) with a hierarchical clustering tree showing that combinatorials clustered according to the groups. **E.** box plots for peak heights for the three groups (singletons – red, combinatorials - green, hotspots - blue) for T cells and macrophages.

A Chip sequencing experiment typically identifies thousands to hundreds of thousands of genome-wide binding sites of a TF in a cell type. In order to investigate if distinct characteristics of peaks grouped based on the number of binding factors at a binding location, we collected genome-wide binding events of transcription-related factors (TFs) across 10 different normal and cancer murine cell types. In each cell type, ChIP sequencing data sets (see Methods for details) for 6 to 21 TFs (108 samples in total) were collected. Peaks were called in each sample (see Methods for details) with the number of peaks ranging from 1,000 to 100,000 in 108 samples (Figure 1B). The number of TFs at binding sites followed an exponential curve such that about half of the regions were bound by only one TF in any given cell type and less than 0.5% occupied by all TFs studied. We divided all peaks in a cell type into three groups: singletons or low-occupancy, peaks bound by only one transcription-related factor (Figure 1A), combinatorials or mid-occupancy, peaks bound by a combination of transcription-related factors (Figure 1A) and hotspots or high-occupancy, peaks bound by more than five TFs studied in a given cell type (Figure 1A, Additional file 1: Table S31). On average about 50% of all the peaks in a cell type were singletons and combinatorials while only 0.1-2% was classified as hotspots (Figure 1C). We assigned peaks in these three groups to genomic locations: promoter, 5′ UTR, 3′ UTR, exon, intron or inter-genic using HOMER [7]. The location of peaks followed a uni-modal distribution with a peak at the TSS (Additional file 2: Figure S1). Singleton peaks were specifically under-represented in promoter regions (P value: 5.2e-3) and 5′ UTR regions (P value: 8.9e-3) (Additional file 2: Figure S2).

In order to investigate the overlap of the three groups across cell types, we constructed a hierarchical tree of 30 peak sets (three groups each in 10 cell types). To construct hierarchical tree, we first integrated of all 108 peak sets across 10 cell types resulted in 408,003 unique peaks. We then constructed a binary matrix of 408,003 rows and 30 columns recording the presence or absence of peaks in each group for each cell type and built hierarchical tree using Pearson’s correlation coefficient as a distance measure. Many samples clustered according to the group across multiple cell types (Figure 1D). The peaks in three groups in a given cell type are mutually exclusive by definition but the fact that combinatorials across many cell types cluster according to the groups is a notable observation. This suggests that each genomic location is marked for occupancy i.e. mid-occupied loci in one cell type were more likely to be bound by multiple factors rather than one factor in another cell type. Hotspots of related cell types cluster into two tight clusters. The two embryonic stem (ES) cell samples were the most similar to each other as expected. The four related blood cell types – HPC, Erythroid, MK progenitors and B cells formed second separate dense cluster of hotspot peaks.

In *D. melanogaster*, peaks occupied at high levels by transcription factors *in vivo* drive patterned gene expression, whereas those occupied only at lower levels mostly do not [6]. We calculated average peak height for the three groups where peak height was defined as the total number of tags in a 400 bp window around the peak summit (see Methods). Singletons consistently showed significantly lower peak height compared to combinatorials and hotspots (Additional file 1: Table S32). For example, the average peak height of Fli1 bound singleton regions in T cells is significantly lower than combinatorials (P value < 1e-323) as were the average peak heights of Cebpβ bound regions in macrophages where singletons were significantly lower (P value < 1e-323) than combinatorials (Figure 1E).

Finally, we collected ChIP sequencing data for Oct4 in ES cells generated by three independent labs [8-10] and found about 1000 Oct4 peaks overlapping in all three experiments. The 1000 peaks were enriched for combinatorials and hotspots but were depleted for singleton regions (Additional file 2: Figure S3). Combinatorials and hotspot binding events were found consistently across different experiments.

Taken together, singletons were depleted in promoter and 5’ UTR regions, had lower peak height and were not consistently found across multiple experiments.

### Hotspots preferentially bind in cell type-specific gene neighbourhoods

**Figure 2:**
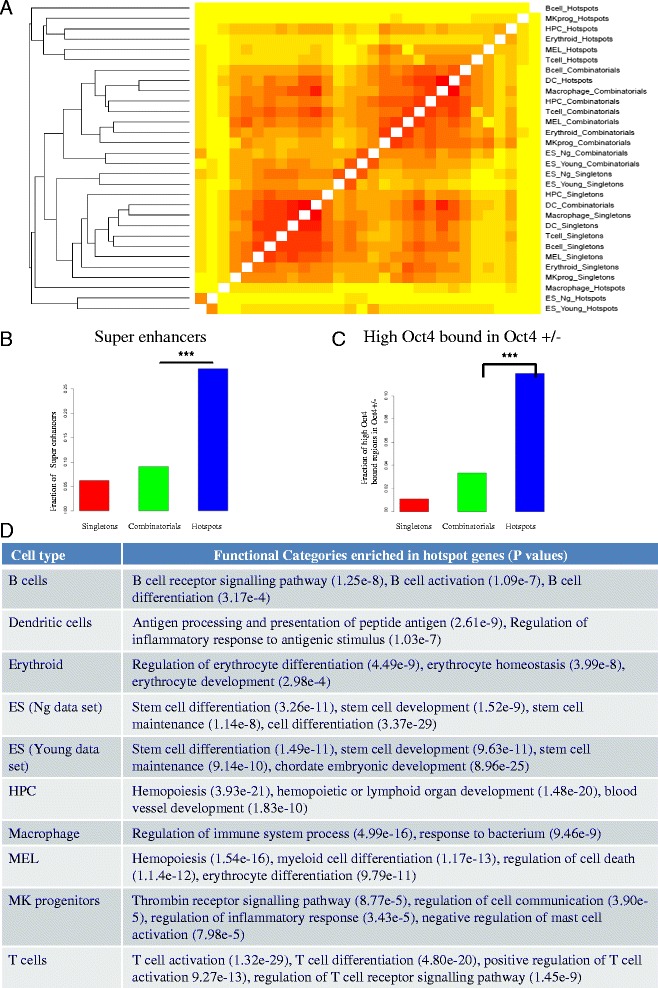
**Hotspots preferentially bind in cell type-specific gene neighbourhoods. A**. A heatmap of Pearson’s correlation coefficients of genes in 30 sets (three groups of 10 cell types) with a hierarchical clustering tree showing that singletons and combinatorials clustered together in a tight cluster while hotspot gene sets were very distinct from each other. **B**. Fraction of ES super enhancers defined by Whyte *et al.* [11] in three groups showing hotspots significantly overlapped with super enhancers. **C**. Fraction of high Oct4 bound regions in Oct4+/− compared to Oct4+/+ [12] ES cells in three groups showing hotspots enriched for high Oct4 bound regions in Oct4+/− and also pluripotent genes **D**. Functional enrichments of hotspot genes in 10 cell types along with p values showing that hotspot genes were enriched for cell type specific genes.

The hierarchical clustering of 30 peak sets clustered them largely according to occupancy. To investigate if the group-wise clustering of peaks is also reflected at a gene level, we mapped all peaks to the nearest gene resulting into a total of 21,609 genes. We created a binary gene matrix of 21,609 rows and 30 columns and built a hierarchical tree using Pearson’s correlation coefficient as a distance measure. In contrast to the peak overlap, the gene overlap clustered all singleton and combinatorial peaks together forming one cluster while hotspot genes showed a lower overlap across cell types ( an exception of the two ES cell samples, Figure 2A). By considering peaks only in promoters and performing a hierarchical clustering, the three groups clustered largely together and combinatorials formed one tight cluster (Additional file 2: Figure S4). Thus, combinatorials were located in the vicinity of similar genes across cell types but hotspots were present near a distinct set of genes in each sample. The functional enrichment of hotspot genes highlighted enrichment for cell type specific functions (Figure 2D), such as B cell hotspots which were over-represented for the B cell receptor signalling pathway (P value: 1.25e-8) and B cell activation (P value: 1.09e-7), while stem cell hotspots were enriched for stem cell differentiation (1.49e-11) and stem cell development (9.63e-11). Though binding events of all peaks were enriched for cell type specific functions, hotspot genes had a much higher cell type specific functional enrichment than all peaks in a cell type.

Recently, three groups generated a list of cell type specific enhancers in mouse ES cells using diverse experiments. Whyte *et al.* [11] showed that master transcription factors such as Oct, Sox, and Nanog form ‘super-enhancers’ at key cell identity genes that span large domains and drive cell-type-specific gene expression program. We calculated the overlap of ES cell peaks in all three groups with super-enhancers and noted that about 30% of super-enhancers overlapped with hotspots in ES cells (Figure 2B, P value:2.7e-8). Karwacki-Neisius *et al.* [12] showed that *Oct4+/−* (reduced Oct4 expression) ES cells have increased Oct4 binding at pluripotency enhancers. Only the enhancer regions strongly bound by Oct4 in *Oct4+/−* compared to *Oct4+/+* had a preferential bias towards hotspot regions (Figure 2C, P value: 4.61e-29). As Oct4 binding in *Oct4+/−* was shown to be specifically enriched near pluripotent genes, this confirmed that hotspots occupied cell type specific gene loci. Stadler *et al.* [13] generated a methylome map in murine ES cells and noted that low methylated regions mark multi-TF bound enhancer regions in the vicinity of cell type specific genes. The overlap of high- and low-methylated regions with three groups in ES cells confirmed that hotspots were specifically depleted for high-methylated and enriched for low-methylated regions in ES cells (Additional file 2: Figure S6). Taken together, by comparing the three groups against three complementary experimental datasets we concluded that hotspots lie preferentially in the vicinity of cell type specific genes.

### Hotspots are enriched for sequence motifs of multiple transcription factors and are preferentially bound by pioneering factors

In order to search for properties distinguishing the three groups, we calculated the average mammalian conservation score for the genome sequences of peaks in all three groups. There was no statistically significant difference in the fraction of conserved peaks between the three groups (Additional file 1: Table S33). In *C. elegans,* hotspot-associated genes were more likely to be essential and were enriched for a variety of functions such as growth, reproduction, and larval and embryonic development [4]. To check if this observation holds true in the mammalian system, we downloaded essential genes in mouse [14] and investigated whether any of the three groups were preferentially bound near essential genes. Though there was a relatively smaller fraction of essential genes in singletons compared to hotspots, the difference was not statistically significant (Additional file 1: Table S34). Thus, genomic conservation and gene essentiality did not distinguish the three groups.

In *D. melanogaster,* hotspots were enriched for sequence motifs of many but not all TFs under study and those without sequence motif enrichment were shown to bind via protein-protein interaction [2]. They were enriched for sequence motifs of two global activators Zelda and GAGA and this sequence signature alone is sufficient to distinguish the hotspot regions [3]. They were also enriched for BEAF-32 and Trl/GAF insulator motifs [15]. In *C. elegans, cis*-regulatory sequences of TFs are not always present in hotspots, the binding was associated with open chromatin [4]. In line with this, hotspots were enriched for ‘GAGA’ sequence motifs [16]. On the other hand, the depletion of *cis*-regulatory motifs was observed in human hotspots where the sequence motif for TFs in ‘cold’ regions were more than two- fold more enriched than in hotspot regions and no ‘GAGA’ or similar motif was found [5], but they mentioned the possible presence of other sequence-specific motifs. Indeed Foley *et al.* found that on average, any given HOT promoter contained about four different types of motifs [17]. In summary there is no evidence so far regarding a specific *cis*-regulatory signature in hotspot peaks in mammals, nor is there a consensus whether hotspots are enriched or depleted for TF sequence motifs.

**Figure 3:**
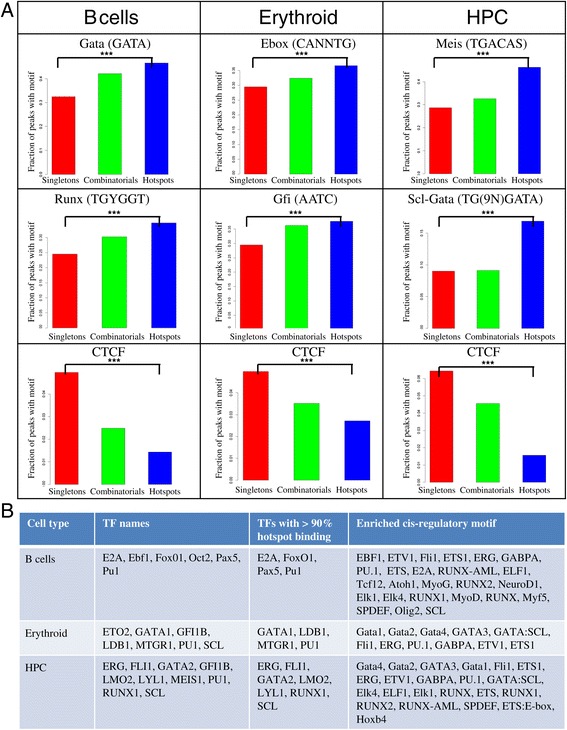
**Hotspots are enriched for sequence motifs of multiple transcription factors. A**. Bar graphs of fraction of peaks with a given sequence motif in the three groups (singletons – red, combinatorials - green, hotspots - blue) with the name of the sequence motif along with the sequence in IUPAAC format for three representative motifs for each of B cells, erythroid and HPCs (see Additional file 1: Figure S1 for the complete list of motifs). **B**. A table of cell type, transcription-related factors analysed by ChIP sequencing, transcription-related factors binding to more than 90% of hotspot peaks and statistically significant known sequence motifs found in hotspots for B cells, erythroid and HPCs (see Additional file 1: Figure S2 for the complete table with 10 cell types).

To check if any sequence motif signature discriminates the three groups, we calculated the number of occurrences of mammalian *cis*-regulatory sequence motifs and compared them between the three groups. In most cell types, the motifs associated with the transcription factors known to be key players for that cell type were significantly enriched in hotspot regions compared to singletons. For example, Gata (7.0e-2) and Runx (1.94e-8) motifs were enriched in B cell hotspots, E-box (6.26e-8) and Gfi1 (3.00e-3) motifs were enriched in Erythroid hotspots while Meis (1.58e-17) and Scl-Gata (2.44e-13) motifs were enriched in HPC hotspots (Figure 3A, Additional file 1: Table S35). In contrast, there was a significant depletion of some motifs in hotspots compared to singletons. In particular, the CTCF motif was less abundant in hotspots across multiple cell types (Figure 3A). In summary, hotspots were enriched for many known transcription factor motifs (Figure 3B) including the one not characterized by ChIP sequencing such as HOX motif in HPCs (Additional file 1: Table S37). As GC content can bias the motif enrichment, we confirmed that the GC content across three groups was not significantly different across ten cell types (Additional file 1: Table S36). Moreover, we validated that the motif enrichment was consistent only when the promoter peaks were considered in three groups (Additional file 1: Figure S5).

Siersbaek *et al.* identified ~1,000 TF hotspots during adipogenesis and demonstrated that Cebpβ marks a large number of these hotspots and is required for chromatin remodeling during differentiation [18]. This hypothesizes that pioneering TFs in a cell type mark hotspots. We identified TFs preferentially binding in hotspots (more than 90% regions bound) and found Pu.1 marking B cell hotspots and Cebps marking macrophage hotspots (Figure 3B and Additional file 1: Table S37). Med12 was bound at more than 99% of ES hotspots which proposes hotspots to be under control of mediator complex [11]. Thus hotspots regions were indeed bound by pioneering factors across multiple cell types. On the other hand, only around 1% of peaks of Suz12 overlapped with hotspots (under-representation p value < 0.05). As Suz12 predominantly acts as a transcriptional repressor this suggests that hotspots might act as transcriptional hubs mainly for activation.

Altogether, murine hotspots did not contain a signature sequence motif but were enriched for motifs of many transcription factors including pioneering factors which mainly act as transcription activators.

### Hotspots lie near highly expressed genes and are enriched for enhancers in nucleosome flanked regions

**Figure 4:**
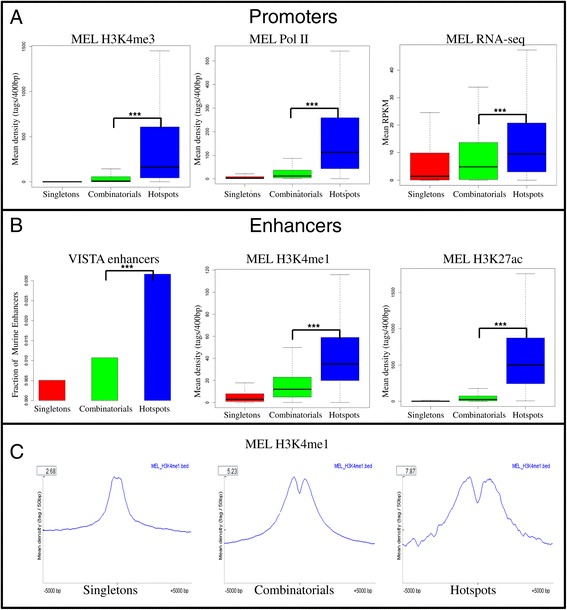
**Hotspots lie near highly expressed genes and are enriched for enhancers in nucleosome flanked regions. A**. Boxplots for the peak height for H3K4me3, RNA polymerase II using ChIP sequencing as well as average RPKM value using RNA sequencing data in MEL cells for the three groups (singletons – red, combinatorials - green, hotspots - blue) showing higher peak height for all chromatin modifications in hotspots. **B**. Bar graph of fraction of VISTA enhancers [19] and Boxplots for the peak height for H3K4me1, H3K27ac using ChIP sequencing in the three groups in MEL cells concluding hotspots were enriched for enhancers. **C**. The average density plot of MEL H3K4me1 in the 10 kb window for each of the three groups showing hotspots had a bi-modal H3K4me1 peak while singletons have a mono-modal peak.

In order to investigated whether each group shows a distinct chromatin signature, we collected data for three activating chromatin modifications in enhancers and promoters (H3K27ac, H3K4me1, H3K4me3), CCCTC-binding factor (CTCF), RNA polymerase II (PolII) binding as well as RNA sequencing data in murine erythroleukemia (MEL) cells from the ENCODE resource. Hotspot peaks showed significantly higher binding in all six datasets compared to the other two groups. In *D. melanogaster*, hotspot genes were expressed at higher levels during early embryogenesis but the difference is reduced later in development [2]. In humans, using ENCODE data, it was shown that genes associated with ‘hot’ regions were expressed at higher levels [4]. In line with this, H3K4me3, PolII binding as well as gene expression measured in RPKM values using RNA sequencing data (Figure 4A) in MEL cells were significantly higher in hotspots (P values: 1.49e-116, 2.40e-162 and1.9e-20 respectively).

Pennachio *et al.* [20] predicted about 5,500 high-confidence murine tissue-specific enhancers for 61 tissue types by integrating tissue-specific expression data, conservation information and *cis*-regulatory motifs and further experimentally validated them using transgenic assays in mouse (VISTA enhancers). MEL hotspots were enriched in VISTA enhancers compared to the singletons and combinatorials (Figure 4B, P value: 4.64e-6). Hotspots in other cell types were also enriched for VISTA enhancers (Additional file 1: Table S38). MEL hotspots had significantly higher H3K4me1 as well as H3K27ac (P values 5.81e-120 ad 2.63e-275 respectively). Histone H3K4me1 marks for enhancer regions whereas histone H3K27ac distinguishes active enhancers from inactive/poised enhancer elements containing H3K4me1 alone [21]. Hotspots therefore mark active enhancer regions in a given cell type.

Hoffman *et al.* [22] showed high nucleosome positioning at the center of bimodal H3K4me1 peaks compared to mono-modal in mouse islets and liver. The average density plots of H3K4me1 showed a very strong bimodal distribution at hotspots while singletons showed a mono-modal distribution (Figure 4C). This suggests that multiple transcription-related factors can indeed displace nucleosomes for strong enhancer activity at hotspot regions. Hotspots were therefore enriched for nucleosome flanked multiple TF-bound loci. As expected, genes associated with flanked TF-bound loci were more abundantly expressed than those associated with nucleosomal loci, consistent with flanked sites being active enhancer elements [23].

In order to validate these observations in another cell type, we collected genome-wide chromatin modification data sets in ES cells. Similar to MEL cells [24], all activating chromatin modifications as well as expression values in RPKM using RNA sequencing data showed significant enrichment in hotspots (Additional file 2: Figure S6). The repressive chromatin modifications such as H3K27me3 were depleted in hotspots (Additional file 2: Figure S6). Taken together, we integrated genome wide chromatin binding patterns with TF ChIP sequencing data to show that hotspot regions were enriched for enhancer regions which are located near highly expressed cell type specific genes. Furthermore, the H3K4me1 profile showed a strong bimodal peak at hotspots suggesting nucleosome displacement for transcription activation.

### ES hotspots are preferentially occupied by activators and hotspots regions and might be under *Setdb1* control for silencing

**Figure 5:**
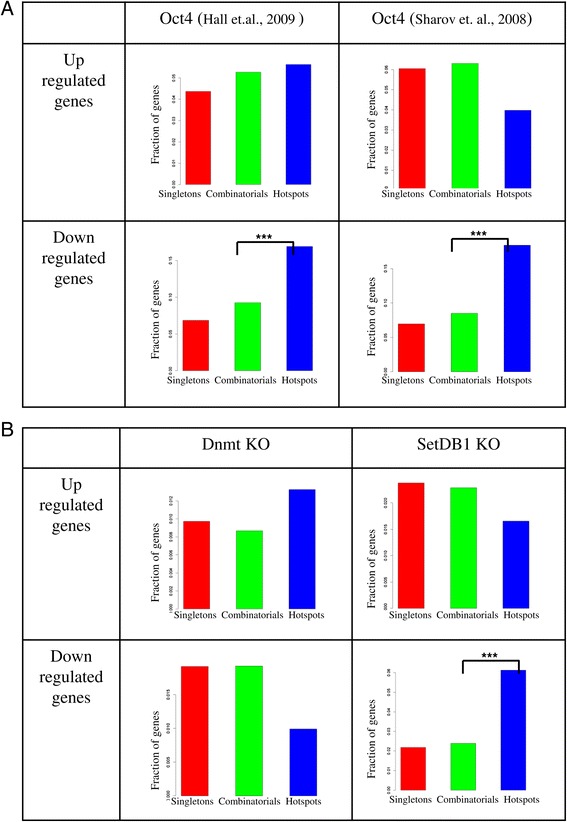
**ES hotspots are preferentially occupied by activators. A**. Overlap of genes in three groups in ES Ng dataset with up- and down-regulated genes upon Oct4 knockdown in ES cells showing hotspots were enriched for only down-regulated genes. **B**. Fraction of up- and down-regulated genes upon Dnmt and Set1db knock out in three groups in ES Ng dataset showing hotspot enrichment only in Set1db KO down-regulated genes.

In order to establish the functional relevance of the binding in three groups, we collected a range of differentially expressed gene sets after TF perturbations in ES cells. Firstly we collected differentially expressed genes after *Oct4* knockdown from two independent experiments [25,26]. ES hotspot genes were specifically enriched for down-regulated genes (P value 3.23e-17) after *Oct4* knockdown but not up-regulated genes (Figure 5A). This suggests that Oct4 binds to hotspot regions to act as a transcriptional activator.

Nishiyama *et al.* [27] identified potential functional targets by identifying differentially expressed genes after overexpressing 50 TFs in ES cells. ES hotspot genes were over-represented for differentially expressed regulators such as *Etv3* (8.28e-3). However, ES hotspot genes were under-represented (Additional file 3: Figure S7) in differentially expressed genes after overexpression of repressors such as *Atf3* (6.62e-3), *Gadd45a* (6.62e-3), *Mybl2* (1.65e-3), *Rhox6* (1.65e-3) and *Tcf4* (4.97e-3). This corroborates the previous observation that hotspot regions were predominantly occupied by activators of transcription (Additional file 1: Table S39).

Not only transcription factors but also other chromatin modifiers such as *Baf250* [28], a subunit of the BAF chromatin remodelling complex, as well as *Hdac1* [29], a histone deacetylase involved in the removal of acetyl groups from core histones, were preferentially bound at ES hotspots (Additional file 1: Table S40). Enrichment in hotspot loci suggests Hdac1 might act as a transcriptional activator. Though HDACs are generally considered repressors of transcription, it has been noted recently that *Hdac1* can indeed act as an activator [29].

As hotspots mark highly active enhancers driving cell type specific gene expression, these regions need to be silenced in other cell types. Karimi *et al.* [30] showed DNA methylation and *Setdb1* methylation work on different sets of genes, where only 7% of the genes up-regulated in a *Setdb1* knockout also were up-regulated in *Dnmt* knock out in ES cells. No significant difference was observed between singletons, combinatorial or hotspot genes for differentially expressed genes after Dnmt knock out (Figure 5B). Challen *et al.* [31] found ~1,500 loci under *Dnmt3a* control in HPCs. There was no bias in *Dnmt3* occupied loci in HPC, neither towards CpG islands in the three groups. However, genes differentially expressed after *Setdb1* knock out were enriched for hotspots (Figure 5B) suggesting that hotspots might be regulated by Setdb1.

Altogether, hotspots might be used by activators to control expression of neighbouring genes as they were enriched for differentially expressed genes after perturbation of activators in ES cells. Furthermore, we suggested that ES hotspots might be under *Setdb1* control for silencing.

## Conclusion

The combinatorial action of multiple TFs controls regulation of gene expression where TFs activate or repress gene expression via binding to *cis*-regulatory sequence elements in gene loci. Identification of functional target genes regulated by specific transcription factors is critical for understanding of the molecular mechanisms behind transcriptional control. Multi-TF ChIP sequencing studies have led to a remarkable observation that there were regions in the genome bound by almost all of the factors, the so called ‘Transcriptional Hotspots’ [1-4]. These regions acted as active enhancers of developmentally essential genes [3]. On the other hand, low occupancy regions did not drive reporter gene expression [6] questioning the functional significance of these peaks.

In order to study whether there are indeed classes of peaks with distinct characteristics based on their occupancy, we collected 108 TF binding ChIP sequencing datasets in 10 murine cell types. All peaks in a cell type were classified into three groups based on the number of factors bound: singletons (low occupancy), combinatorials (mid occupancy) and hotspots (high occupancy). Singletons were depleted in promoters and 3’ UTR, had a low peak height and were less conserved across multiple biological replicates putting in doubt their functional relevance. On the other hand, conserved cis-regulatory sequences were thought be more functionally relevant but the three groups could not be distinguished with respect to sequence conservation. Indeed, it has been shown in yeast that the low affinity protein-DNA interactions were conserved as well as being functionally relevant [32].

The hierarchical clustering of 30 peak sets (three groups each in 10 cell types) clustered combinatorials according to occupancy. Putative target gene sets of singletons and combinatorial peaks clustered into one tight cluster whereas each hotspot gene set consisted of unique set of genes with cell type specific functional gene enrichment. The presence of hotspot regions near cell type specific genes has previously been observed in many cell types across species (e.g. Siersbaek *et al.* [18])*.* We have shown previously that most TF peaks cluster according to the cell type [33].

GAGA and Zelda signature sequence motifs were associated with hotspots in *C. elegans* and *D. melanogaster* [16]. We did not find a signature sequence motif for any of the groups but hotspots were enriched for sequence motifs of many key transcription factors as well as for pioneering factors in a cell type. Hotspots were specifically depleted for CTCF sequence motif across all cell types. Though depleted in CTCF sequence motifs compared to singletons, the fraction of CTCF peaks and their mean peak height at hotspots is higher (data not shown). We further intersected three groups with diverse chromatin marks and concluded that hotspots were enriched for enhancers present in the vicinity of highly expressed genes. Moreover, we identified a chromatin modification that can distinguish hotspots from singletons. Bimodal H3K3me1 peaks were specifically associated with hotspots, and mono-modal peaks with singletons. This suggests that three events, binding by multiple factors, creation of a nucleosome-depleted region at the binding site and establishment of bivalent H3K4me1 marks go hand in hand.

We so far confirmed that hotspots had strong occupancy (high peak height) and were enriched for cell type specific enhancers but their functional relevance is still a puzzle. There are various theories such as it has been suggested that they might serve as sinks for TFs [34]. In humans, as hotspots were depleted for TF motifs, hotspots were thought to be highly open chromatin marks where TFs bind non-specifically. Contrary to this, we find hotspots enriched for TF motifs especially for pioneering cell type specific factors such as Pu.1 in B cells. Pu.1 binding has been shown to initiate nucleosome modeling followed by H3K4 mono-methylation [7]. Our hypothesis therefore is that pioneering factors establish stable open chromatin regions at hotspots by recruiting specific chromatin marks which in turn facilitates binding of additional TFs at their motifs or non-specifically.

Finally, as hotspots are cell type-specific active enhancer regions, they need to be silenced in other cell types. Hotspot genes in ES cells significantly overlapped with genes perturbed after *Setdb1* knockout and not *Dnmt* knockout suggesting that Setdb1 might play a role in ES hotspot silencing.

## Methods

### Data collection and classification

Genome wide binding patterns of transcription-related factors were obtained for five blood lineages (B cells, erythroid, haematopoietic progenitor cells (HPC), macrophage and T cells) [35], two embryonic stem cells [36], dendritic cells [37], MEL cells [38]. Peaks were called, with default parameters, using the peak calling program MACS [39] using an input control if provided in the original study. In total 108 peak sets were collected across 10 cell types (Additional file 1: Table S31). The binding events (peaks) of all transcription-related factors in a given cell type were merged to produce the total number of peaks in each cell type. These peaks were then divided into three groups according to the occupancy. To divide the regulatory peaks into three groups, we defined singletons as the genomic regions bound by one factor only in a cell type; hotspots were defined as genomic regions bound by more than five transcription-related factors. The choice of criterion for defining hotspots is discussed in detail in Additional file 3. Combinatorials were then defined as peaks which did not belong to either of the two groups. Additional file 3: Tables S1–30 are the peaks in BED format using mm9 genome assembly for all 30 peak sets (three peak lists corresponding to the three groups in each cell type).

### Clustering, overlap and enrichment calculation

Genomic location bias such as promoters, 5’ UTR, 3’UTR, exon and intron as well as known and *de-novo cis*-regulatory sequence motif enrichments and functional enrichments were calculated using HOMER [7]. The promoter was defined as -1 kb to +100 bp region around transcription start site. Mammalian conservation tracks were downloaded from the UCSC genome browser. To calculate peak height, each peak was made of uniform width 400 bp and the total number of tags in the 400 bp window was used as a proxy for peak height. UCSC gene annotation was used to map peaks to genes. Peaks present in the promoter or gene body were assigned to the corresponding gene and inter-genic peaks were assigned to the nearest gene within 50 kb (both upstream and downstream). The total of unique genome-wide peak locations was 408,002 which mapped to 21,607 unique genes. A binary peak matrix of 408,002 rows and 30 columns (three groups in 10 cell types) was created where ‘1’ represents presence of a peak and ‘0’ the absence. Similarly, a gene matrix of 21,607 rows and 30 columns was created. Hierarchical clustering of peak and gene matrices was performed using Pearson’s correlation coefficient as a similarity measure and complete linkage. All boxplots and bar charts were generated using R. Genomic sequences of the peaks were downloaded using UCSC galaxy tool and the occurrences of *cis*-regulatory sequence motifs were obtained using TFBSseach [40]. The percentage of total hotspot peaks occupied by each transcription-related factor in a given cell type was calculated and TFs with more than 90% hotspot binding were tabulated in Figure 4 (Additional file 2: Table S37 for full list). Statistical significance of the differences between groups was calculated using the Mann Whitney U test.

### Additional datasets

CpG islands in mouse were downloaded from CpG_MI [41]. Methylation density plots for the three groups using H3K4me1 data in MEL cells were generated using SeqMiner [42]. Experimentally validated tissue specific enhancers (VISTA enhancers) in mouse were obtained from VISTA [19]. The lists of differentially expressed genes upon overexpression of transcription factors in embryonic stem (ES) cells were obtained from the data of Nishiyama *et al.* [27]. ChIP sequencing data for chromatin modifications in ES cells was obtained from Teif *et al.* [24] whereas methylation profiling in ES cells was obtained from Stadler *et al.* [13]. The list of super-enhancers in ES cells were downloaded from Whyte *et al.* [11] and the genomic regions differentially occupied in Oct4+/− compared to Oct4+/+ were downloaded from Karwacki-Neisius *et al.* [12]. Differentially expressed genes at multiple time points after Oct4 knockdown were obtained from Hall *et al.* [25] and Sharov *et al.* [26]. Differentially expressed genes after Baf250 and Hdac1 were obtained from Gao *et al.* [28] and Zupkovitz *et al.* [29]. For consistency the differentially expressed gene lists after perturbation of transcription factors in ES cells were compared against both the ES Ng dataset (Figure 5) and the ES Young dataset (Additional file 1: Figure S6). The RNA sequencing data for ES cells is available at GEO (GSE42152).

### Availability of supporting data

This study was purely analysis of existing public data sets. The raw data used in this study is available at GEO with accession numbers GSE11431, GSE31233, GSE11724, GSE36104, GSE36030, GSE22178, GSE21314, GSE33913, GSE12346, GSE21512, GSE35024, GSE21978, GSE21614, GSE30142 and GSE29193.

## Additional files

Additional file 1:
**Supplementary data and supplementary tables 31-40.**
Additional file 2:
**Supplementary figures.**
Additional file 3:
**Supplementary tables 1:30.**

## References

[1] Wilson NK, Foster SD, Wang X, Knezevic K, Schütte J, Kaimakis P, Chilarska PM, Kinston S, Ouwehand WH, Dzierzak E, Pimanda JE, de Bruijn MFTR, Göttgens B (2010). Combinatorial transcriptional control in blood stem/progenitor cells: genome-wide analysis of ten major transcriptional regulators. Cell Stem Cell.

[2] Moorman C, Sun LV, Wang J, de Wit E, Talhout W, Ward LD, Greil F, Lu X-J, White KP, Bussemaker HJ, van Steensel B (2006). Hotspots of transcription factor colocalization in the genome of Drosophila melanogaster. Proceedings of the National Academy of Sciences.

[3] Kvon EZ, Stampfel G, Yanez-Cuna JO, Dickson BJ, Stark A (2012). HOT regions function as patterned developmental enhancers and have a distinct cis-regulatory signature. Genes & Development.

[4] Gerstein MB, Lu ZJ, Van Nostrand EL, Cheng C, Arshinoff BI, Liu T, Yip KY, Robilotto R, Rechtsteiner A, Ikegami K, Alves P, Chateigner A, Perry M, Morris M, Auerbach RK, Feng X, Leng J, Vielle A, Niu W, Rhrissorrakrai K, Agarwal A, Alexander RP, Barber G, Brdlik CM, Brennan J, Brouillet JJ, Carr A, Cheung M-S, Clawson H, Contrino S (2010). Integrative analysis of the Caenorhabditis elegans genome by the modENCODE project. Science.

[5] Yip KY, Cheng C, Bhardwaj N, Brown JB, Leng J, Kundaje A, Rozowsky J, Birney E, Bickel P, Snyder M, Gerstein M (2012). Classification of human genomic regions based on experimentally determined binding sites of more than 100 transcription-related factors. Genome Biology.

[6] Fisher WW, Li JJ, Hammonds AS, Brown JB, Pfeiffer BD, Weiszmann R, MacArthur S, Thomas S, Stamatoyannopoulos JA, Eisen MB, Bickel PJ, Biggin MD, Celniker SE (2012). DNA regions bound at low occupancy by transcription factors do not drive patterned reporter gene expression in Drosophila. Proc. Natl. Acad. Sci. U.S.A..

[7] Heinz S, Benner C, Spann N, Bertolino E, Lin YC, Laslo P, Cheng JX, Murre C, Singh H, Glass CK (2010). Simple combinations of lineage-determining transcription factors prime cis-regulatory elements required for macrophage and B cell identities. Mol. Cell.

[8] Ang Y-S, Tsai S-Y, Lee D-F, Monk J, Su J, Ratnakumar K, Ding J, Ge Y, Darr H, Chang B, Wang J, Rendl M, Bernstein E, Schaniel C, Lemischka IR (2011). Wdr5 mediates self-renewal and reprogramming via the embryonic stem cell core transcriptional network. Cell.

[9] Chen X, Xu H, Yuan P, Fang F, Huss M, Vega VB, Wong E, Orlov YL, Zhang W, Jiang J, Loh Y-H, Yeo HC, Yeo ZX, Narang V, Govindarajan KR, Leong B, Shahab A, Ruan Y, Bourque G, Sung W-K, Clarke ND, Wei C-L, Ng H-H (2008). Integration of external signaling pathways with the core transcriptional network in embryonic stem cells. Cell.

[10] Marson A, Levine SS, Cole MF, Frampton GM, Brambrink T, Johnstone S, Guenther MG, Johnston WK, Wernig M, Newman J, Calabrese JM, Dennis LM, Volkert TL, Gupta S, Love J, Hannett N, Sharp PA, Bartel DP, Jaenisch R, Young RA (2008). Connecting microRNA genes to the core transcriptional regulatory circuitry of embryonic stem cells. Cell.

[11] Whyte WA, Orlando DA, Hnisz D, Abraham BJ, Lin CY, Kagey MH, Rahl PB, Lee TI, Young RA (2013). Master transcription factors and mediator establish super-enhancers at key cell identity genes. Cell.

[12] Karwacki-Neisius V, Göke J, Osorno R, Halbritter F, Ng JH, Weiße AY, Wong FCK, Gagliardi A, Mullin NP, Festuccia N, Colby D, Tomlinson SR, Ng H-H, Chambers I (2013). Reduced Oct4 expression directs a robust pluripotent state with distinct signaling activity and increased enhancer occupancy by Oct4 and Nanog. Cell Stem Cell.

[13] Stadler MB, Murr R, Burger L, Ivanek R, Lienert F, Schöler A, van Nimwegen E, Wirbelauer C, Oakeley EJ, Gaidatzis D, Tiwari VK, Schübeler D (2011). DNA-binding factors shape the mouse methylome at distal regulatory regions. Nature.

[14] Liao B-Y, Zhang J (2007). Mouse duplicate genes are as essential as singletons. Trends in Genetics.

[15] Roy S, Ernst J, Kharchenko PV, Kheradpour P, Negre N, Eaton ML, Landolin JM, Bristow CA, Ma L, Lin MF, Washietl S, Arshinoff BI, Ay F, Meyer PE, Robine N, Washington NL, Di Stefano L, Berezikov E, Brown CD, Candeias R, Carlson JW, Carr A, Jungreis I, Marbach D, Sealfon R, Tolstorukov MY, Will S, Alekseyenko AA, Artieri C, The modENCODE Consortium (2010). Identification of Functional Elements and Regulatory Circuits by Drosophila modENCODE. Science.

[16] Farley E, Levine M (2012). HOT DNAs: a novel class of developmental enhancers. Genes & Development.

[17] Foley JW, Sidow A (2013). Transcription-factor occupancy at HOT regions quantitatively predicts RNA polymerase recruitment in five human cell lines. BMC Genomics.

[18] Siersbæk R, Nielsen R, Mandrup S (2012). Transcriptional networks and chromatin remodeling controlling adipogenesis. Trends in Endocrinology & Metabolism.

[19] Visel A, Minovitsky S, Dubchak I, Pennacchio LA (2007). VISTA enhancer browser–a database of tissue-specific human enhancers. Nucleic Acids Research.

[20] Pennacchio LA, Ahituv N, Moses AM, Prabhakar S, Nobrega MA, Shoukry M, Minovitsky S, Dubchak I, Holt A, Lewis KD, Plajzer-Frick I, Akiyama J, De Val S, Afzal V, Black BL, Couronne O, Eisen MB, Visel A, Rubin EM (2006). In vivo enhancer analysis of human conserved non-coding sequences. Nature.

[21] Creyghton MP, Cheng AW, Welstead GG, Kooistra T, Carey BW, Steine EJ, Hanna J, Lodato MA, Frampton GM, Sharp PA, Boyer LA, Young RA, Jaenisch R (2010). From the Cover: Histone H3K27ac separates active from poised enhancers and predicts developmental state. P Natl Acad Sci.

[22] Hoffman BG, Robertson G, Zavaglia B, Beach M, Cullum R, Lee S, Soukhatcheva G, Li L, Wederell ED, Thiessen N, Bilenky M, Cezard T, Tam A, Kamoh B, Birol I, Dai D, Zhao Y, Hirst M, Verchere CB, Helgason CD, Marra MA, Jones SJM, Hoodless PA (2010). Locus co-occupancy, nucleosome positioning, and H3K4me1 regulate the functionality of FOXA2-, HNF4A-, and PDX1-bound loci in islets and liver. Genome Research.

[23] Zhang X, Robertson G, Woo S, Hoffman BG, Gottardo R (2012). Probabilistic inference for nucleosome positioning with MNase-Based or Sonicated Short-Read Data. PLoS ONE.

[24] Teif VB, Vainshtein Y, Caudron-Herger M, Mallm J-P, Marth C, Höfer T, Rippe K (2012). Genome-wide nucleosome positioning during embryonic stem cell development. Nat. Struct. Mol. Biol..

[25] Hall J, Guo G, Wray J, Eyres I, Nichols J, Grotewold L, Morfopoulou S, Humphreys P, Mansfield W, Walker R, Tomlinson S, Smith A (2009). Oct4 and LIF/Stat3 additively induce Krüppel factors to sustain embryonic stem cell self-renewal. Cell Stem Cell.

[26] Sharov AA, Masui S, Sharova LV, Piao Y, Aiba K, Matoba R, Xin L, Niwa H, Ko MSH (2008). Identification of Pou5f1, Sox2, and Nanog downstream target genes with statistical confidence by applying a novel algorithm to time course microarray and genome-wide chromatin immunoprecipitation data. BMC Genomics.

[27] Nishiyama A, Xin L, Sharov AA, Thomas M, Mowrer G, Meyers E, Piao Y, Mehta S, Yee S, Nakatake Y, Stagg C, Sharova L, Correa-Cerro LS, Bassey U, Hoang H, Kim E, Tapnio R, Qian Y, Dudekula D, Zalzman M, Li M, Falco G, Yang H-T, Lee S-L, Monti M, Stanghellini I, Islam MN, Nagaraja R, Goldberg I, Wang W (2009). Uncovering early response of gene regulatory networks in ESCs by systematic induction of transcription factors. Cell Stem Cell.

[28] Gao X, Tate P, Hu P, Tjian R, Skarnes WC, Wang Z (2008). ES cell pluripotency and germ-layer formation require the SWI/SNF chromatin remodeling component BAF250a. Proceedings of the National Academy of Sciences.

[29] Zupkovitz G, Tischler J, Posch M, Sadzak I, Ramsauer K, Egger G, Grausenburger R, Schweifer N, Chiocca S, Decker T, Seiser C (2006). Negative and positive regulation of gene expression by mouse histone deacetylase 1. Mol. Cell. Biol..

[30] Karimi MM, Goyal P, Maksakova IA, Bilenky M, Leung D, Tang JX, Shinkai Y, Mager DL, Jones S, Hirst M, Lorincz MC (2011). DNA methylation and SETDB1/H3K9me3 regulate predominantly distinct sets of genes, retroelements, and chimeric transcripts in mESCs. Cell Stem Cell.

[31] Challen GA, Sun D, Jeong M, Luo M, Jelinek J, Berg JS, Bock C, Vasanthakumar A, Gu H, Xi Y, Liang S, Lu Y, Darlington GJ, Meissner A, Issa J-PJ, Godley LA, Li W, Goodell MA (2012). Dnmt3a is essential for hematopoietic stem cell differentiation. Nat. Genet..

[32] Tanay A (2006). Extensive low-affinity transcriptional interactions in the yeast genome. Genome Res..

[33] Hannah R, Joshi A, Wilson NK, Kinston S, Göttgens B (2011). A compendium of genome-wide hematopoietic transcription factor maps supports the identification of gene regulatory control mechanisms. Exp. Hematol..

[34] MacArthur S, Li X-Y, Li J, Brown JB, Chu HC, Zeng L, Grondona BP, Hechmer A, Simirenko L, Keränen SV, Knowles DW, Stapleton M, Bickel P, Biggin MD, Eisen MB (2009). Developmental roles of 21 Drosophila transcription factors are determined by quantitative differences in binding to an overlapping set of thousands of genomic regions. Genome Biology.

[35] Joshi A, Hannah R, Diamanti E, Göttgens B (2013). Gene set control analysis predicts hematopoietic control mechanisms from genome-wide transcription factor binding data. Exp. Hematol.

[36] Martello G, Sugimoto T, Diamanti E, Joshi A, Hannah R, Ohtsuka S, Göttgens B, Niwa H, Smith A (2012). Esrrb is a pivotal target of the Gsk3/Tcf3 axis regulating embryonic stem cell self-renewal. Cell Stem Cell.

[37] Garber M, Yosef N, Goren A, Raychowdhury R, Thielke A, Guttman M, Robinson J, Minie B, Chevrier N, Itzhaki Z, Blecher-Gonen R, Bornstein C, Amann-Zalcenstein D, Weiner A, Friedrich D, Meldrim J, Ram O, Cheng C, Gnirke A, Fisher S, Friedman N, Wong B, Bernstein BE, Nusbaum C, Hacohen N, Regev A, Amit I (2012). A high-throughput chromatin immunoprecipitation approach reveals principles of dynamic gene regulation in mammals. Mol. Cell.

[38] Project Consortium ENCODE (2011). A user’s guide to the encyclopedia of DNA elements (ENCODE). PLoS Biol..

[39] Zhang Y, Liu T, Meyer CA, Eeckhoute J, Johnson DS, Bernstein BE, Nussbaum C, Myers RM, Brown M, Li W, Liu XS (2008). Model-based analysis of ChIP-Seq (MACS). Genome Biology.

[40] Chapman MA, Donaldson IJ, Gilbert J, Grafham D, Rogers J, Green AR, Göttgens B (2004). Analysis of multiple genomic sequence alignments: a web resource, online tools, and lessons learned from analysis of mammalian SCL loci. Genome Res..

[41] Su J, Zhang Y, Lv J, Liu H, Tang X, Wang F, Qi Y, Feng Y, Li X (2009). CpG_MI: a novel approach for identifying functional CpG islands in mammalian genomes. Nucleic Acids Research.

[42] Ye T, Krebs AR, Choukrallah M-A, Keime C, Plewniak F, Davidson I, Tora L (2011). seqMINER: an integrated ChIP-seq data interpretation platform. Nucleic Acids Res..

