# Supplementary material for: Mammalian transcriptional hotspots are enriched for tissue specific enhancers near cell type specific highly expressed genes and are predicted to act as transcriptional activator hubs
Source: BMC Bioinformatics. 2014 Dec 30;15(1):412. doi: 10.1186/s12859-014-0412-0 (PMC4302108; doi:10.1186/s12859-014-0412-0)
Supplement: Additional file 1: — Supplementary data and supplementary tables 31-40. [file 12859_2014_412_MOESM1_ESM.docx]

Supplementary Table and Figure legends for

**Mammalian transcriptional hotspots are enriched for tissue specific enhancers near cell type specific highly expressed genes and are predicted to act as transcriptional activator hubs.**

Anagha Joshi^1,^*

^1^ The Roslin Institute, University of Edinburgh, Easter Bush Campus, Midlothian, EH25 8GR, UK

**Supplementary methods:**

**Choice of criterion for defining hotspots:** Transcription factors have been shown to collaborate with each other for transcription control in mammalian system. The genome wide binding patterns of multiple transcription factors surprisingly found genomic regions occupied by very many factors and were called transcriptional hotspots. We collected genome-wide binding patterns of multiple transcription factors across 10 murine cell types to study the characteristics of hotspots across multiple cell types. As the number of factors studied in this collection is different in each cell type and same factors are not studied across multiple cell types, choosing a criterion to define hotspots is not trivial. Moreover, all ChIP sequencing samples were not sequenced to same depth adding yet another layer of factor to consider. We performed random permutations of binding profiles of all TFs studied in each cell type and observed that the number of regions bound by more than 5 factors was much higher (statistically significant) in real data compared to random and therefore used the criterion for 5 or more factors to define hotspots across multiple cell types. This definition of hotspots indeed suffers from not being independent of factors studied i.e. more the number of transcription factors studied, the higher the number of regions likely to be defined as hotspots. Most other criteria to define hotspots will have the same bias indeed. For example, % of transcription factors bound (e.g. 75% factors bound) is another way of defining hotspots but the number of hotspots defined by this criterion will show a strong anti-correlation with the number of factor studied by ChIP sequencing. Specifically, 75% factors bound definition classifies only 9 (Ng dataset) and 100 (Young dataset) peaks as hotspots in ES cells where more than 10 factors were studied and over 5000 regions in cell types with fewer factors studied such as Macrophages. Moorman *et. al.* (2006)[1] first observed hotspots as genomic regions where very many transcription factors bind than expected by chance. Our more than 5 criterion is consistent with this idea and therefore our choice for this study.

References:

1. Moorman C, Sun LV, Wang J, de Wit E, Talhout W, Ward LD, Greil F, Lu X-J, White KP, Bussemaker HJ, van Steensel B: **Hotspots of transcription factor colocalization in the genome of Drosophila melanogaster**. *Proc Natl Acad Sci* 2006, **103**:12027–12032.

**Supplementary Tables:**

Supplementary tables 1-30 are provided as bed files of 30 peak lists (peak lists for the three groups – Singletons, Combinatorials and Hotspots in 10 cell types). The name of each peak file specifies the cell type and the group e.g. Bcell_hotspots.bed provides genomic co-ordinated of hotspot regions in B cells using mm9 genome assembly.

**Supplementary Table 31:** The number of peaks for each group in ten cell types B.

|  | **all peaks** | **Singletons** | **Combinatorials** | **Hotspots** |
| --- | --- | --- | --- | --- |
| Dendritic cells | 237,882 | 130,558 | 71,022 | 36,302 |
| Erythroid | 45,803 | 26,746 | 18,323 | 734 |
| ES (Ng data set) | 75,230 | 53,412 | 21,215 | 603 |
| ES (Young data set) | 60,405 | 26,196 | 31,327 | 2,882 |
| HPC | 54,460 | 26,101 | 24,745 | 3,614 |
| Macrophage | 89,968 | 48,892 | 40,635 | 441 |
| MEL | 81,556 | 47,600 | 30,483 | 3473 |
| MK progenitors | 26,074 | 14,044 | 11,896 | 134 |
| T cells | 89,895 | 43,595 | 42,478 | 3,822 |
| B cells | 78,915 | 55,363 | 23,425 | 127 |

**Supplementary Table 32: peak height.** Average peak height as well as pairwise comparisons of P values of the statistical significance of the difference in average peak height in the three groups for each transcription ChIP sequencing data across 10 cell types

| **#** | **TF (cell type)** | **Mean singletons** | **Mean combinatorials** | **Mean hotspots** | **S vs C** | **S vs H** | **C vs H** |
| --- | --- | --- | --- | --- | --- | --- | --- |
| 1 | Erg ( HPC ) | 12.8 | 31.1 | 70.5 | 0 | 0 | 0 |
| 2 | Fli1 ( HPC ) | 6.7 | 14.2 | 50.5 | 0 | 0 | 0 |
| 3 | Gata2 ( HPC ) | 9.7 | 10.8 | 38.7 | 1.12e-9 | 2.42e-275 | 5.97e-287 |
| 4 | Gfi1b ( HPC ) | 6.9 | 9.7 | 30.0 | 0 | 3.26e-295 | 7.62e-205 |
| 5 | Lmo2 ( HPC ) | 5.6 | 9.2 | 51.3 | 1.20e-100 | 0 | 0 |
| 6 | Lyl1 ( HPC ) | 5.0 | 6.6 | 32.9 | 0.01 | 0 | 0 |
| 7 | Meis1 ( HPC ) | 7.5 | 10.2 | 25.4 | 2.12e-235 | 3.47e-303 | 3.19e-251 |
| 8 | Pu1 ( HPC ) | 6.4 | 8.8 | 11.9 | 5.00e-257 | 3.58e-69 | 1.13e-20 |
| 9 | Runx1 ( HPC ) | 2.9 | 6.3 | 23.6 | 0 | 0 | 0 |
| 10 | Scl ( HPC ) | 6.4 | 9.7 | 44.4 | 0 | 0 | 0 |
| 11 | Eto2 (Erythroid) | 7.8 | 16.9 | 105.7 | 1.39e-37 | 1.70e-129 | 1.76e-114 |
| 12 | Gata1 (Erythroid) | 5.1 | 16.6 | 51.4 | 9.27e-277 | 5.27e-237 | 2.19e-152 |
| 13 | Ldb1 (Erythroid) | 6.3 | 20.1 | 89.7 | 1.83e-105 | 7.77e-230 | 1.38e-194 |
| 14 | Mtgr1 (Erythroid) | 2.5 | 5.8 | 29.9 | 1.72e-79 | 2.10e-162 | 1.39e-133 |
| 15 | Tal1 (Erythroid) | 6.7 | 16.8 | 70.7 | 1.81e-91 | 7.68e-205 | 5.86e-173 |
| 16 | Gfi1b (Erythroid) | 11.5 | 15.1 | 45.9 | 8.38e-172 | 4.45e-27 | 2.80e-4 |
| 17 | Pu1 (MEL) | 5.7 | 12.5 | 16.4 | 0 | 1.90e-39 | 0.03 |
| 18 | Fli1 (T cells) | 5.9 | 13.8 | 35.7 | 0 | 6.94e-54 | 0.16 |
| 19 | Gata3 (T cells) | 6.8 | 10.4 | 25.8 | 3.28e-200 | 2.04e-33 | 5.08e-5 |
| 20 | Stat3 (T cells) | 5.3 | 9.8 | 18.6 | 0 | 0.29 | 2.39e-28 |
| 21 | Stat4 (T cells) | 7.1 | 24.5 | 57.5 | 0 | 1.32e-20 | 0.03 |
| 22 | Stat5a (T cells) | 1.1 | 2.5 | 5.2 | 0 | 2.55e-18 | 0.01 |
| 23 | Stat6 (T cells) | 5.9 | 17.8 | 41.8 | 0 | 3.03e-28 | 0.19 |
| 24 | Tbet (T cells) | 16.2 | 47.0 | 96.8 | 0 | 7.72e-21 | 5.50e-4 |
| 25 | Pu1 (T cells) | 16.3 | 14.7 | 31.8 | 0 | 3.10e-138 | 6.43e-31 |
| 26 | E2A (B cells) | 3.8 | 14.3 | 41.3 | 0 | 2.55e-40 | 3.67e-5 |
| 27 | Ebf (B cells) | 10.4 | 16.9 | 52.9 | 1.32e-20 | 0.48 | 0.05 |
| 28 | FoxO1 (B cells) | 17.4 | 6.8 | 22.9 | 9.92e-181 | 1.33e-18 | 0.05 |
| 29 | Oct2 (B cells) | 1.1 | 1.4 | 3.6 | 7.75e-75 | 5.49e-8 | 0.37 |
| 30 | Pu1 (B cells) | 27.9 | 35.0 | 172.7 | 0 | 1.84e-21 | 0.02 |
| 31 | Pax5 (B cells) | 16.9 | 71.1 | 96.1 | 3.33e-300 | 2.50e-4 | 1.72e-6 |
| 32 | Cebpa (Macrophages) | 2.6 | 14.6 | 38.8 | 5.71e-262 | 5.83e-29 | 7.75e-3 |
| 33 | Cebpb (Macrophages) | 2.9 | 16.3 | 43.3 | 0 | 7.1e-29 | 0.10 |
| 34 | Pparg (Macrophages) | 4.5 | 6.6 | 19.5 | 1.17e-195 | 1.05e-15 | 0.75 |
| 35 | P65 (Macrophages) | 10.7 | 27.6 | 50.5 | 0 | 2.25e-7 | 2.59e-5 |
| 36 | Pu1 (Macrophages) | 3.2 | 4.3 | 4.3 | 2.00e-19 | 4.1e-3 | 0.57 |
| 37 | Stat1 (Macrophages) | 14.4 | 43.8 | 147.8 | 0 | 4.90e-26 | 0.04 |

**Supplementary Table 33: Mammalian conservation fraction**

| **Cell types** | **Singletons** | **Combinatorials** | **Hotspots** |
| --- | --- | --- | --- |
| B cells | 0.62 | 0.75 | 0.73 |
| Dendritic cells | 0.54 | 0.61 | 0.99 |
| Erythroid | 0.68 | 0.80 | 0.77 |
| ES (Ng. et. al) | 0.61 | 0.75 | 0.58 |
| ES (Young et. al.) | 0.61 | 0.64 | 0.52 |
| HPC | 0.66 | 0.78 | 0.70 |
| Macrophage | 0.65 | 0.70 | 0.55 |
| MEL | 0.58 | 0.68 | 0.88 |
| MK progenitors | 0.67 | 0.85 | 1 |
| T cells | 0.62 | 0.68 | 0.88 |

**Supplementary Table 34: Essential gene fraction**

| **Cell types** | **Singletons** | **Combinatorials** | **Hotspots** |
| --- | --- | --- | --- |
| B cells | 0.09 | 0.10 | 0.11 |
| Dendritic cells | 0.09 | 0.10 | 0.12 |
| Erythroid | 0.08 | 0.08 | 0.11 |
| ES (Ng. et. al) | 0.09 | 0.08 | 0.09 |
| ES (Young et. al.) | 0.09 | 0.09 | 0.08 |
| HPC | 0.09 | 0.08 | 0.12 |
| Macrophage | 0.09 | 0.10 | 0.11 |
| MEL | 0.09 | 0.09 | 0.09 |
| MK progenitors | 0.09 | 0.08 | 0.11 |
| T cells | 0.10 | 0.11 | 0.10 |

**Supplementary Table 35:** Mean number of motifs in each of the three groups together with the pairwise p values calculated using Wilcoxon rank sum test (last 3 columns) 10 sequence motifs (AATC, CANNTG, GATA, GGAW, SSYAAY, TG(9N)GATA, TGACAS, TGYGGT and TTCNNNGAA) in the three groups for HPCs, B cells and Erythroids..

| **Cell type** | **Motif** | **Mean singletons** | **Mean combina** | **Mean hotspots** | **S vs C** | **S vs H** | **C vs H** |
| --- | --- | --- | --- | --- | --- | --- | --- |
| HPC | AATC | 1.5 | 1.5 | 1.7 | 2.48e-46 | 6.18e-18 | 1.85e-7 |
| HPC | CANNTG | 1.7 | 1.6 | 1.9 | 0.95 | 1.82e-16 | 5.22e-21 |
| HPC | GATA | 1.2 | 1.3 | 1.8 | 5.10e-15 | 6.52e-60 | 1.84e-54 |
| HPC | GGAW | 5.9 | 5.7 | 5.0 | 1.17e-126 | 2.22e-23 | 1.32e-4 |
| HPC | SSYAAY | 1.2 | 1.3 | 1.4 | 1.47e-85 | 9.62e-13 | 0.04 |
| HPC | TG(9N)GATA | 0.1 | 0.1 | 0.2 | 0.99 | 2.44e-13 | 2.80e-13 |
| HPC | TGACAS | 0.5 | 0.5 | 0.6 | 8.77e-4 | 1.58e-17 | 8.97e-15 |
| HPC | TGYGGT | 0.3 | 0.4 | 0.5 | 1.53e-8 | 1.25e-10 | 8.29e-7 |
| HPC | TTCNNNGAA | 0.2 | 0.2 | 0.2./com | 3.02e-29 | 2.82e-7 | 0.05 |
| B cells | AATC | 1.8 | 1.6 | 1.7 | 0.01 | 0.04 | 0.07 |
| B cells | CANNTG | 1.7 | 2.4 | 2.5 | 2.03e-12 | 5.65e-40 | 5.09e-21 |
| B cells | GATA | 1.4 | 1.3 | 1.6 | 8.92e-12 | 0.07 | 0.73 |
| B cells | GGAW | 5.4 | 5.1 | 5.4 | 1.18e-116 | 6.23e-10 | 0.18 |
| B cells | SSYAAY | 1.3 | 1.3 | 1.1 | 7.19e-86 | 7.20e-5 | 0.74 |
| B cells | TG(9N)GATA | 0.1 | 0.1 | 0.1 | 5.03e-14 | 2.18e-3 | 0.09 |
| B cells | TGACAS | 0.4 | 0.5 | 0.7 | 1.00e-16 | 2.93e-5 | 0.01 |
| B cells | TGYGGT | 0.3 | 0.4 | 0.3 | 2.42e-47 | 1.94e-8 | 0.01 |
| B cells | TTCNNNGAA | 0.2 | 0.2 | 0.1 | 1.70e-54 | 8.25e-4 | 0.92 |
| Erythroid | AATC | 1.6 | 1.6 | 1.7 | 6.82e-5 | 7.78e-4 | 0.01 |
| Erythroid | CANNTG | 1.6 | 1.7 | 1.7 | 6.02e-28 | 6.26e-8 | 7.96e-3 |
| Erythroid | GATA | 1.6 | 1.8 | 1.9 | 8.24e-60 | 8.47e-14 | 3.80e-3 |
| Erythroid | GGAW | 5.2 | 5.5 | 5.8 | 2.31e-34 | 1.02e-10 | 4.71e-4 |
| Erythroid | SSYAAY | 1.2 | 1.3 | 1.4 | 5.61e-54 | 2.22e-7 | 0.42 |
| Erythroid | TG(9N)GATA | 8.2 | 8.2 | 8.3 | 3.02e-81 | 8.93e-9 | 0.80 |
| Erythroid | TGACAS | 8.5 | 8.4 | 8.4 | 4.18e-11 | 3.00e-3 | 0.24 |
| Erythroid | TGYGGT | 8.2 | 8.3 | 8.3 | 1.84e-11 | 0.07 | 0.98 |
| Erythroid | TTCNNNGAA | 8.2 | 8.2 | 8.2 | 4.07e-26 | 1.26e-5 | 0.22 |

**Supplementary Table 36: GC fraction.**

|  | **Mean singletons** | **Mean combinatorials** | **Mean hotspots** |
| --- | --- | --- | --- |
| Dendritic cells | 0.45 | 0.46 | 0.48 |
| Erythroid | 0.49 | 0.50 | 0.51 |
| ES (Ng data set) | 0.50 | 0.54 | 0.51 |
| ES (Young data set) | 0.51 | 0.52 | 0.51 |
| HPC | 0.49 | 0.51 | 0.48 |
| Macrophage | 0.46 | 0.47 | 0.47 |
| MEL | 0.45 | 0.49 | 0.53 |
| MK progenitors | 0.48 | 0.52 | 0.52 |
| T cells | 0.47 | 0.49 | 0.52 |
| B cells | 0.47 | 0.51 | 0.50 |

**Supplementary Table 37:** A list of transcription factors studied by ChIP sequencing, the names of transcription factors with more than 90% binding of hotspot regions as well as the known sequence motifs enriched in hotspots of each cell type for the 10 cell types.

| **#** | **Cell type** | **TF names** | **TFs with > 90% hotspot binding** | **Enriched cis-regulatory motif** |
| --- | --- | --- | --- | --- |
| 1 | B cells | E2A, Ebf1, Fox01, Oct2, Pax5, Pu1, Smad3 | E2A, FoxO1, Pax5, Pu1 | EBF1, ETV1, Fli1, ETS1, ERG, GABPA, PU.1, ETS, E2A, RUNX-AML, ELF1, Tcf12, Atoh1, MyoG, RUNX2, NeuroD1, Elk1, Elk4, RUNX1, MyoD, RUNX, Myf5, SPDEF, Olig2, SCL |
| 2 | Dendritic cells | Ahr, E2f4, Ets2, Hif1a, Irf1, Junb, Egr2, Maff, PU1, Relb, Rela, Stat3, Atf3, Rel, Cebpb, Egr1, Irf2, Irf4, Nfkb1, Stat1, Stat2 | Irf1, Junb, PU1, Rela, Stat3, Atf3, Cebpb, Irf4, Stat1 | Fli1, PU.1, ETS1, GABPA, ERG, Elk4, ETV1, ELF1, ETS, Elk1, Ets1-distal |
| 3 | Erythroid | ETO2, GATA1, GFI1B, LDB1, MTGR1, PU1, SCL | GATA1, LDB1, MTGR1, PU1 | Gata1, Gata2, Gata4, GATA3, GATA:SCL, Fli1, ERG, PU.1, GABPA, ETV1, ETS1 |
| 4 | ES (Ng. et. al) | E2f1, Eset, Esrrb, Klf4, n-Myc, Nanog, Oct4, Sox2, Stat3, Suz12, Tcfcp2l1 | Esrrb | OCT4-SOX2-TCF-NANOG, Sox3, Sox2, Esrrb, Sox6, Oct4, Klf4, Nr5a2, |
| 5 | ES (Young et. al.) | MCAF1, Med12, Med1, Nanog, NelfA, Nipbl, Oct4, REST, Ring1b, Smc1, Smc3, Sox2, Spt5, Suz12, TBP, Tcf3 | Med12, Med1, Nipbl, Oct4 | OCT4-SOX2-TCF-NANOG, Sox3, Oct4, Sox2, Sox6, Oct2, Esrrb, Klf4, Nanog, Erra, Stat3+il23, Stat3, Nanog, STAT4, EKLF, Oct2, Tcfcp2l1, Maz, Erra, Elk4 |
| 6 | HPC | ERG, FLI1, GATA2, GFI1B, LMO2, LYL1, MEIS1, PU1, RUNX1, SCL | ERG, FLI1, GATA2, LMO2, LYL1, RUNX1, SCL | Gata4, Gata2, GATA3, Gata1, Fli1, ETS1, ERG, ETV1, GABPA, PU.1, GATA:SCL, Elk4, ELF1, Elk1, RUNX, ETS, RUNX1, RUNX2, RUNX-AML, SPDEF, ETS:E-box, Hoxb4 |
| 7 | Macrophage | CEBPA, CEBPB, P65, PPARG, PU1, STAT1 | CEBPA, CEBPB, P65, PPARG, PU1, STAT1 | PU.1, CEBP, ETS1, RXR, PPARE, GABPA, ERG, Fli1, HIF1b, ETV1, AP-1, Jun-AP1, CEBP:AP1 |
| 8 | MEL | CMYB, CMYC, CHD2, GATA1, JUND, MAFK, MAX, MXI1, NELFE, SCL, SMC3, TBP, TCF7_EML, USF2 | CMYC, GATA1, MAX, MXI1, TBP, | GATA3, Gata4, Gata1, Gata2, GATA:SCL, GABPA, ETV1, Fli1, PU.1, ERG, ETS1, MYB, EWS:FLI1-fusion, Usf2, ELF1, E-box |
| 9 | MK progenitors | CBFB, ETS1, GATA1, GATA2, RING1B, RUNX1 | CBFB, ETS1, GATA1, GATA2, RING1B, RUNX1 | Gata4, GATA3, Gata1, Gata2, ETS:E-box |
| 10 | T cells | FLI1, GATA3, PU1, STAT3, STAT4, STAT5A, STAT5B, STAT5, STAT6, TBET | FLI1, STAT3, STAT4, STAT5B, STAT5, STAT6, TBET | Fli1, ETS1, ETV1, GABPA, ERG, Elk1, Elk4, Ets1-distal, ELF1, ETS, PU.1, STAT1, STAT5, STAT4, HIF1b, Stat3+il23, Jun-AP1, AP-1, RUNX-AML, RUNX, RUNX1, ETS:RUNX, Stat3, RUNX2, SPDEF, Gata4, GATA3, Gata2, Gata1, STAT6/ |

**Supplementary Table 38: Fraction of VISTA enhancers:** Average enhancer fraction as well as pairwise comparisons of P values of the statistical significance of the difference in average enhancer fraction in the three groups for each transcription ChIP sequencing data across 10 cell types

| # | Cell type | Mean singletons | Mean combinatorials | Mean hotspots | S vs C | S vs H | C vs H |
| --- | --- | --- | --- | --- | --- | --- | --- |
| 1 | B cells | 0.02 | 0.04 | 0.09 | 1.93e-38 | 3.99e-16 | 1.32e-3 |
| 2 | Dendritic cells | 0.01 | 0.02 | 0.13 | 1.90e-15 | 2.11e-251 | 7.24e-122 |
| 3 | Erythroid | 0.03 | 0.04 | 0.10 | 4.99e-5 | 1.76e-11 | 2.86e-6 |
| 4 | ES (Ng. et. al) | 0.02 | 0.04 | 0.06 | 1.35e-28 | 4.09e-4 | 0.49 |
| 5 | ES (Young et. al.) | 0.02 | 0.03 | 0.03 | 0.15 | 0.78 | 0.99 |
| 6 | HPC | 0.02 | 0.04 | 0.06 | 6.81e-11 | 1.46e-3 | 0.29 |
| 7 | Macrophage | 0.02 | 0.03 | 0.06 | 1.06e-14 | 4.61e-6 | 0.02 |
| 8 | MEL | 0.01 | 0.04 | 0.10 | 3.10e-17 | 4.72e-17 | 4.64e-6 |
| 9 | MK progenitors | 0.03 | 0.06 | 0.13 | 6.49e-13 | 4.66e-3 | 0.26 |
| 10 | T cells | 0.01 | 0.03 | 0.07 | 1.02e-9 | 1.31e-35 | 2.27e-16 |

**Supplementary Table 39:** Pairwise comparisons of P values of the statistical significance of the difference in fraction of peaks near differentially expressed genes overexpression of transcription factors within three groups for Ng and Young datasets in ES cells.

| **#** | **Overexpression data** | **Ng dataset** | | | **Young dataset** | | |
| --- | --- | --- | --- | --- | --- | --- | --- |
|  |  | S vs C | S vs H | C vs H | S vs C | S vs H | C vs H |
| 1 | Atf3 | 0.009792 | 0.007691 | 0.006623 | 0.011147 | 0.009972 | 0.006211 |
| 2 | Gadd45a | 0.011252 | 0.008628 | 0.006623 | 0.010498 | 0.01035 | 0.004141 |
| 3 | Mybl2 | 0.003295 | 0.002613 | 0.001656 | 0.003779 | 0.00337 | 0.00207 |
| 4 | Rhox6 | 0.002378 | 0.002219 | 0.001656 | 0.002405 | 0.002441 | 0.00207 |
| 5 | Tcf4 | 0.008537 | 0.006656 | 0.004967 | 0.009582 | 0.00949 | 0.006211 |
| 6 | Etv3 | 0.004774 | 0.003846 | 0.008278 | 0.00565 | 0.004745 | 0.008282 |

**Supplementary Table 40:** Pairwise comparisons of P values of the statistical significance of the difference in fraction of peaks near differentially expressed genes after knock out or knock down of factors within three groups for Ng and Young datasets in ES cells.

| **#** | **KD data** | **Ng dataset** | | | **Young dataset** | | |
| --- | --- | --- | --- | --- | --- | --- | --- |
|  |  | **S vs C** | **S vs H** | **C vs H** | **S vs C** | **S vs H** | **C vs H** |
| 1 | Baf250 | 0.68 | 7.6e-3 | 5.93e-3 | 0.27 | 0.21 | 0.12 |
| 2 | Hdac | 1.39e-8 | 1.48e-8 | 2.99e-5 | 0.38 | 2.25e-9 | 8.37e-9 |
| 3 | Dnmt_down | 0.96 | 0.10 | 0.10 | 0.02 | 0.55 | 0.28 |
| 4 | Dnmt_up | 0.18 | 0.38 | 0.23 | 0.69 | 0.47 | 0.52 |
| 5 | Set1db_down | 0.09 | 7.11e-11 | 6.87e-9 | 0.12 | 3.93e-10 | 7.79e-12 |
| 6 | Set1db_up | 0.47 | 0.25 | 0.30 | 0.81 | 0.15 | 0.13 |
| 7 | Hall_down | 3.59e-28 | 6.07e-22 | 2.65e-10 | 2.10e-3 | 2.50e-14 | 1.73e-11 |
| 8 | Hall_up | 1.39e-7 | 0.13 | 0.70 | 0.55 | 0.03 | 0.04 |
| 9 | Sharov_down | 3.1e-13 | 1.04e-27 | 3.23e-17 | 8.79e-3 | 4.70e-23 | 1.43e-19 |
| 10 | Sharov_up | 0.20 | 0.03 | 0.02 | 0.01 | 0.54 | 0.87 |

**Supplementary Figures**:

**Supp. Figure 1:** The number of peaks as function of distance from TSS.

**Supp. Figure 2:** A bar plot of fraction of peaks in promoters, 3’ UTR, 5’ UTR, exons and introns in three groups (singletons – red, combinatorials- green, hotspots - blue) across 10 cell types.

**Supp. Figure 3:** A. Venn diagram of peaks called by 3 independent Oct4 ChIP sequencing experiments in ES cells from which peaks were classified as identified in all three, identified in only two and identified in only one experiment. B. These three sets were then compared to the three groups in ES Ng and Young datasets to conclude that combinatorials and hotspots are enriched for peaks identified in all three groups while singletons for the one identified in only one group.

**Supp. Figure 4**: A. Heatmap of peaks in promoters in three groups showing that the peaks cluster according to the group. Moreover, combinatorials show highest overlap with each other.

**Supp. Figure 5**: A. Bar graphs of fraction of promoter peaks with a given sequence motif in the three groups (singletons – red, combinatorials - green, hotspots - blue) with the name of the sequence motif along with the sequence in IUPAAC format for three representative motifs for each of B cells, erythroid and HPCs (see supplementary figure 4 for the complete list of motifs). B. A table of cell type and statistically significant known sequence motifs found in hotspots peaks in promoter regions for all 10 cell types.

**Supp. Figure 6:** The difference in average peak height for six datasets (chromatin modifications, CpG methylation and RNA-seq) within three groups for Ng and Young datasets in ES cells.

**Supp. Figure 7:** Similar to Figure 5 but using Young data set to highlight the robustness of results.
