# Supplementary material for: Mammalian transcriptional hotspots are enriched for tissue specific enhancers near cell type specific highly expressed genes and are predicted to act as transcriptional activator hubs
Source: BMC Bioinformatics. 2014 Dec 30;15(1):412. doi: 10.1186/s12859-014-0412-0 (PMC4302108; doi:10.1186/s12859-014-0412-0)
Supplement: Additional file 2: — Supplementary figures. [file 12859_2014_412_MOESM2_ESM.pptx]

## Slide 1
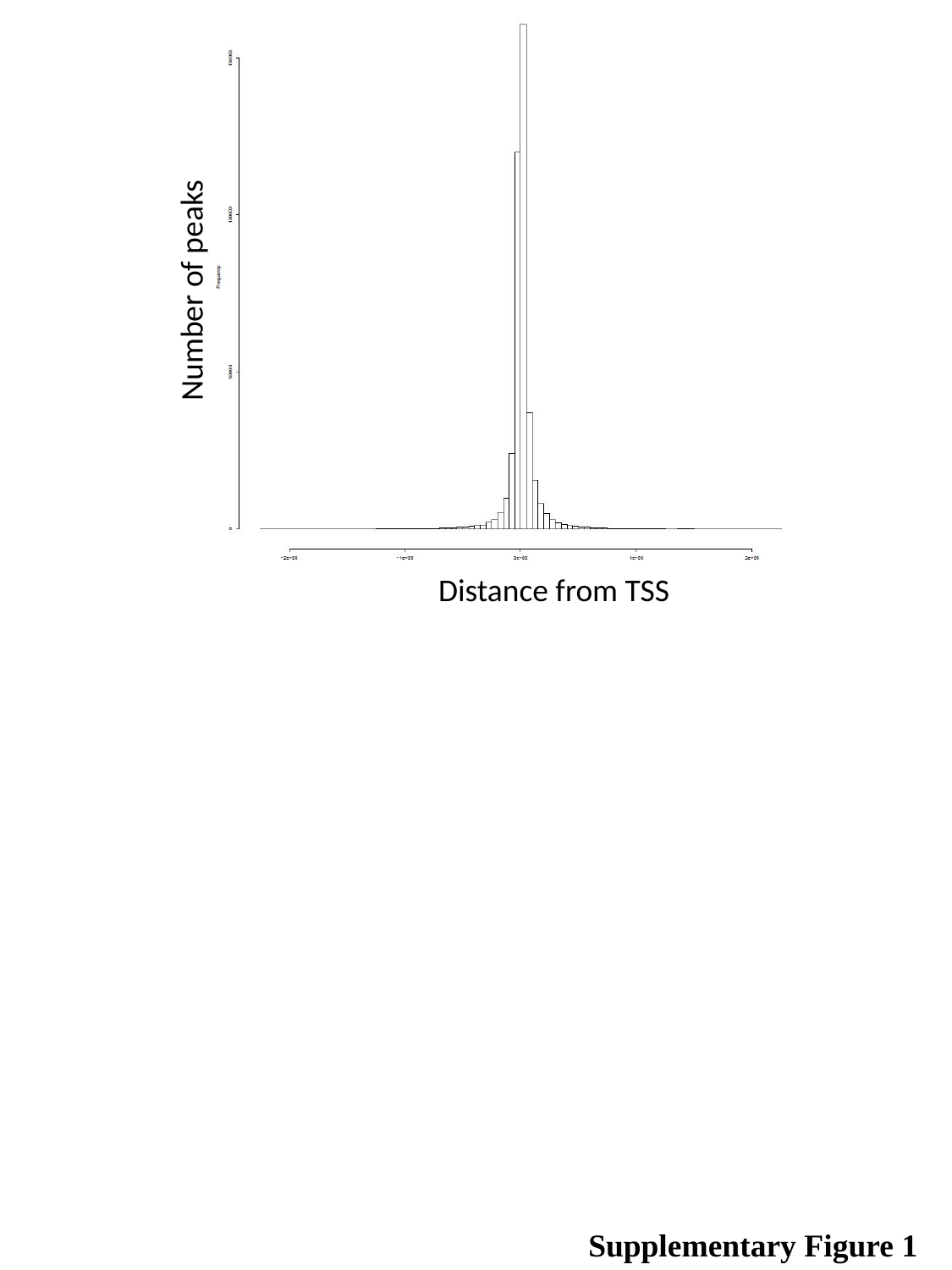

Number of peaks
Distance from TSS
Supplementary Figure 1

## Slide 2
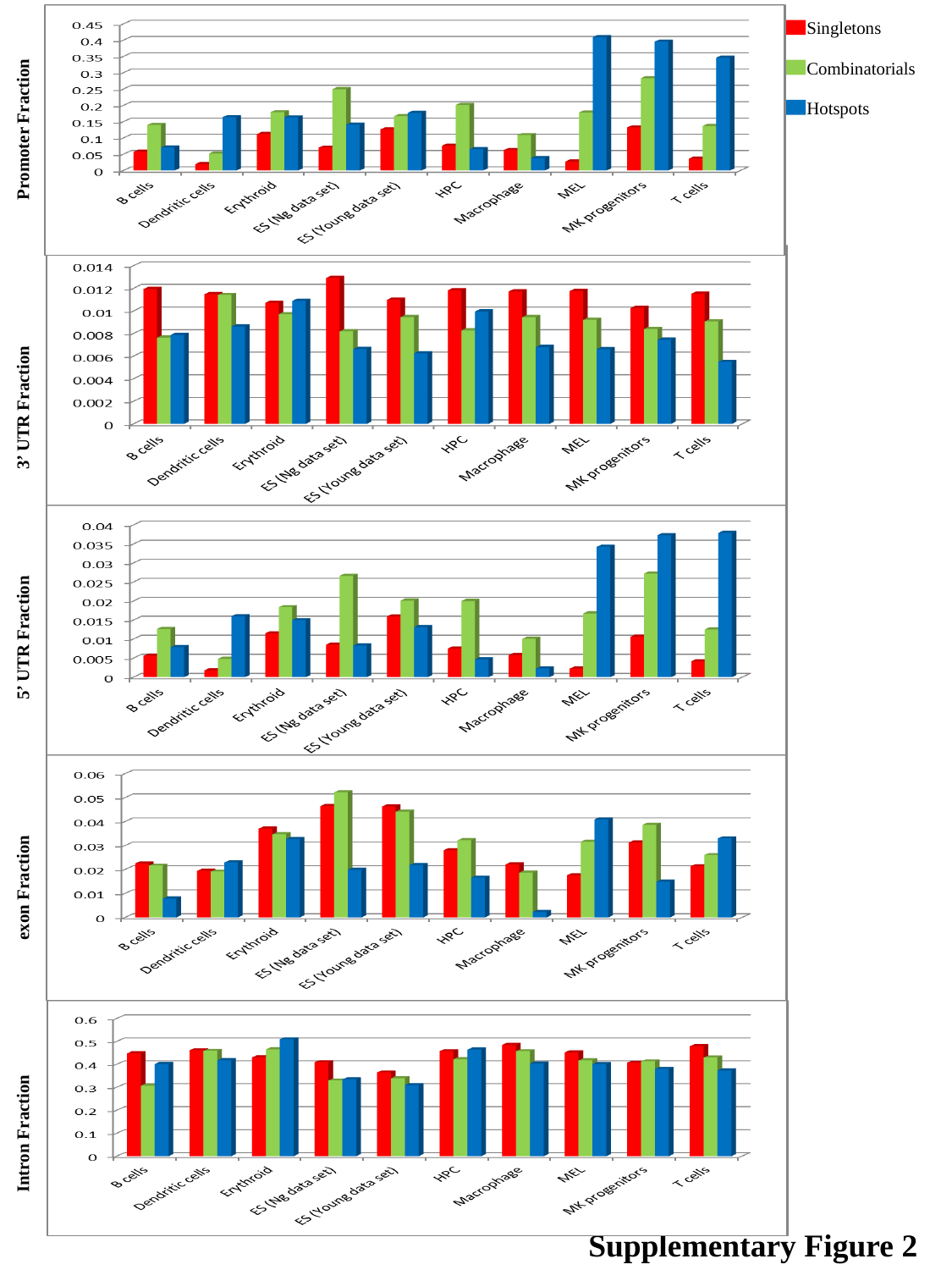

Singletons
Combinatorials
Hotspots
Promoter Fraction
3’ UTR Fraction
5’ UTR Fraction
exon Fraction
Intron Fraction
Supplementary Figure 2

## Slide 3
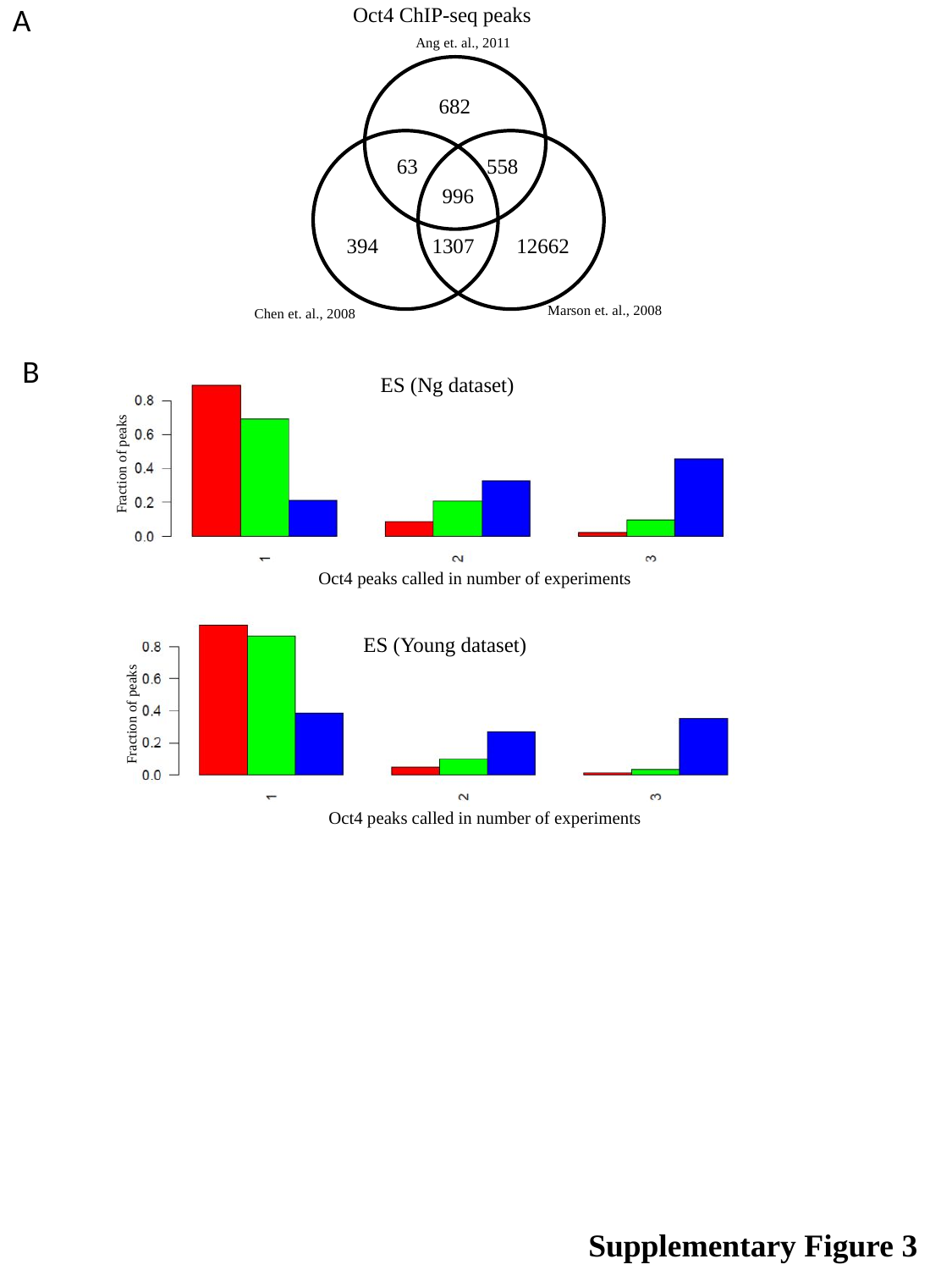

A
Oct4 ChIP-seq peaks
Ang et. al., 2011
682
63
558
996
394
1307
12662
Marson et. al., 2008
Chen et. al., 2008
B
ES (Ng dataset)
Fraction of peaks
Oct4 peaks called in number of experiments
ES (Young dataset)
Fraction of peaks
Oct4 peaks called in number of experiments
Supplementary Figure 3

## Slide 4
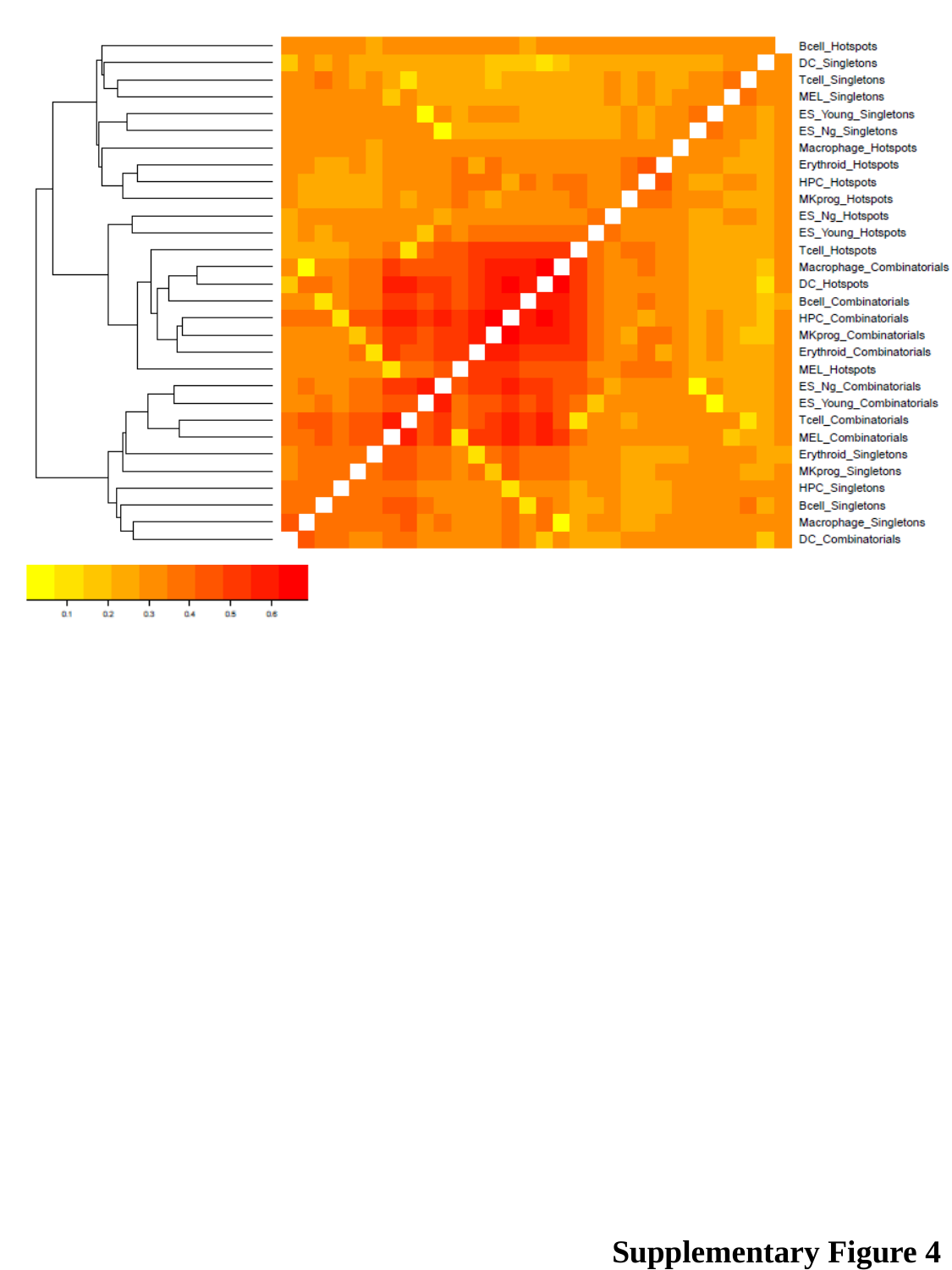

Supplementary Figure 4

## Slide 5
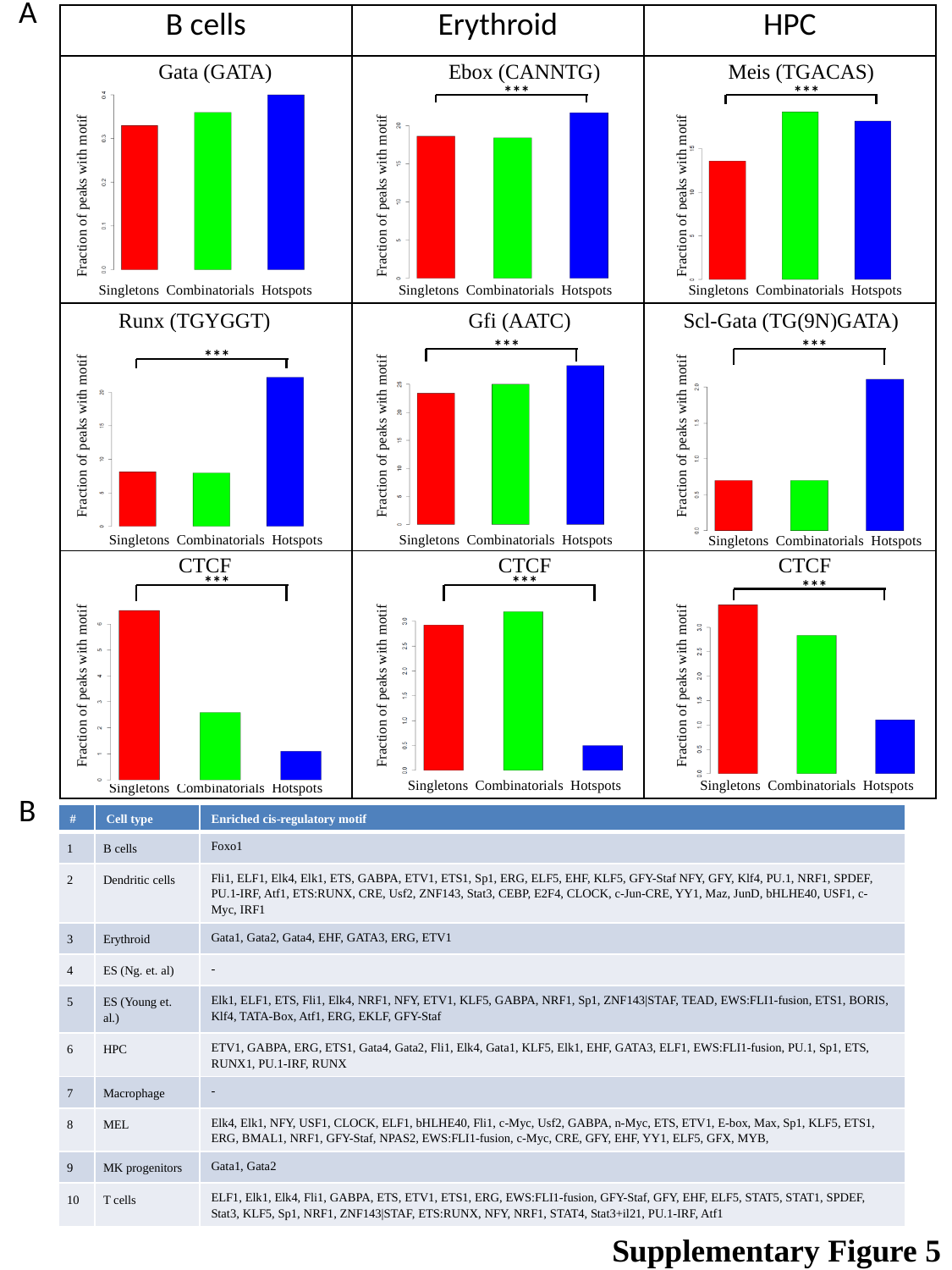

A
| B cells | Erythroid | HPC |
| --- | --- | --- |
| | | |
| | | |
| | | |
Gata (GATA)
Ebox (CANNTG)
Meis (TGACAS)
***
***
Fraction of peaks with motif
Fraction of peaks with motif
Fraction of peaks with motif
Singletons Combinatorials Hotspots
Singletons Combinatorials Hotspots
Singletons Combinatorials Hotspots
Runx (TGYGGT)
Gfi (AATC)
Scl-Gata (TG(9N)GATA)
***
***
***
Fraction of peaks with motif
Fraction of peaks with motif
Fraction of peaks with motif
Singletons Combinatorials Hotspots
Singletons Combinatorials Hotspots
Singletons Combinatorials Hotspots
CTCF
CTCF
CTCF
***
***
***
Fraction of peaks with motif
Fraction of peaks with motif
Fraction of peaks with motif
Singletons Combinatorials Hotspots
Singletons Combinatorials Hotspots
Singletons Combinatorials Hotspots
B
| # | Cell type | Enriched cis-regulatory motif |
| --- | --- | --- |
| 1 | B cells | Foxo1 |
| 2 | Dendritic cells | Fli1, ELF1, Elk4, Elk1, ETS, GABPA, ETV1, ETS1, Sp1, ERG, ELF5, EHF, KLF5, GFY-Staf NFY, GFY, Klf4, PU.1, NRF1, SPDEF, PU.1-IRF, Atf1, ETS:RUNX, CRE, Usf2, ZNF143, Stat3, CEBP, E2F4, CLOCK, c-Jun-CRE, YY1, Maz, JunD, bHLHE40, USF1, c-Myc, IRF1 |
| 3 | Erythroid | Gata1, Gata2, Gata4, EHF, GATA3, ERG, ETV1 |
| 4 | ES (Ng. et. al) | - |
| 5 | ES (Young et. al.) | Elk1, ELF1, ETS, Fli1, Elk4, NRF1, NFY, ETV1, KLF5, GABPA, NRF1, Sp1, ZNF143|STAF, TEAD, EWS:FLI1-fusion, ETS1, BORIS, Klf4, TATA-Box, Atf1, ERG, EKLF, GFY-Staf |
| 6 | HPC | ETV1, GABPA, ERG, ETS1, Gata4, Gata2, Fli1, Elk4, Gata1, KLF5, Elk1, EHF, GATA3, ELF1, EWS:FLI1-fusion, PU.1, Sp1, ETS, RUNX1, PU.1-IRF, RUNX |
| 7 | Macrophage | - |
| 8 | MEL | Elk4, Elk1, NFY, USF1, CLOCK, ELF1, bHLHE40, Fli1, c-Myc, Usf2, GABPA, n-Myc, ETS, ETV1, E-box, Max, Sp1, KLF5, ETS1, ERG, BMAL1, NRF1, GFY-Staf, NPAS2, EWS:FLI1-fusion, c-Myc, CRE, GFY, EHF, YY1, ELF5, GFX, MYB, |
| 9 | MK progenitors | Gata1, Gata2 |
| 10 | T cells | ELF1, Elk1, Elk4, Fli1, GABPA, ETS, ETV1, ETS1, ERG, EWS:FLI1-fusion, GFY-Staf, GFY, EHF, ELF5, STAT5, STAT1, SPDEF, Stat3, KLF5, Sp1, NRF1, ZNF143|STAF, ETS:RUNX, NFY, NRF1, STAT4, Stat3+il21, PU.1-IRF, Atf1 |
Supplementary Figure 5

## Slide 6
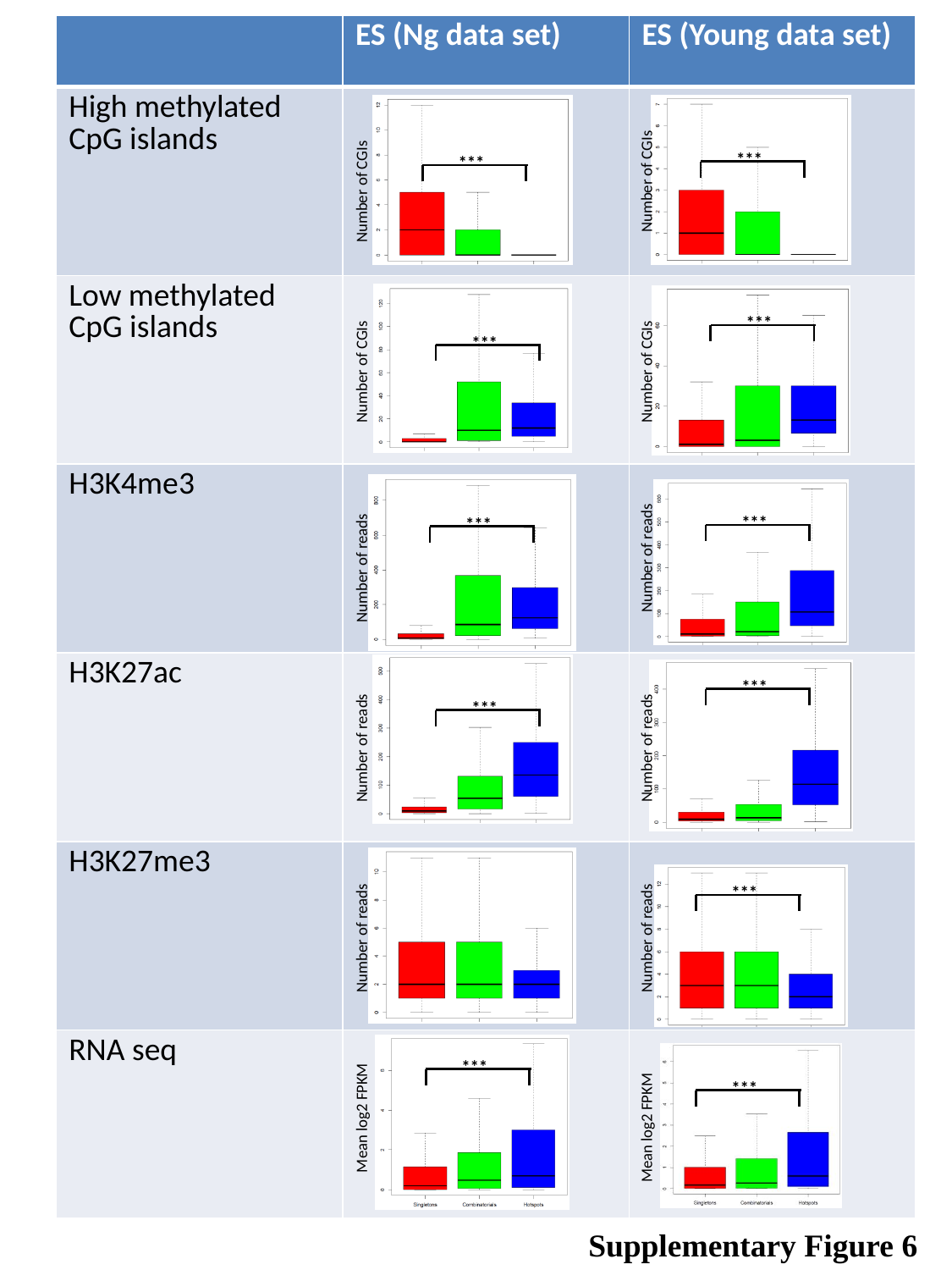

| | ES (Ng data set) | ES (Young data set) |
| --- | --- | --- |
| High methylated CpG islands | | |
| Low methylated CpG islands | | |
| H3K4me3 | | |
| H3K27ac | | |
| H3K27me3 | | |
| RNA seq | | |
***
***
Number of CGIs
Number of CGIs
***
***
Number of CGIs
Number of CGIs
***
***
Number of reads
Number of reads
***
***
Number of reads
Number of reads
***
Number of reads
Number of reads
***
***
Mean log2 FPKM
Mean log2 FPKM
Supplementary Figure 6

## Slide 7
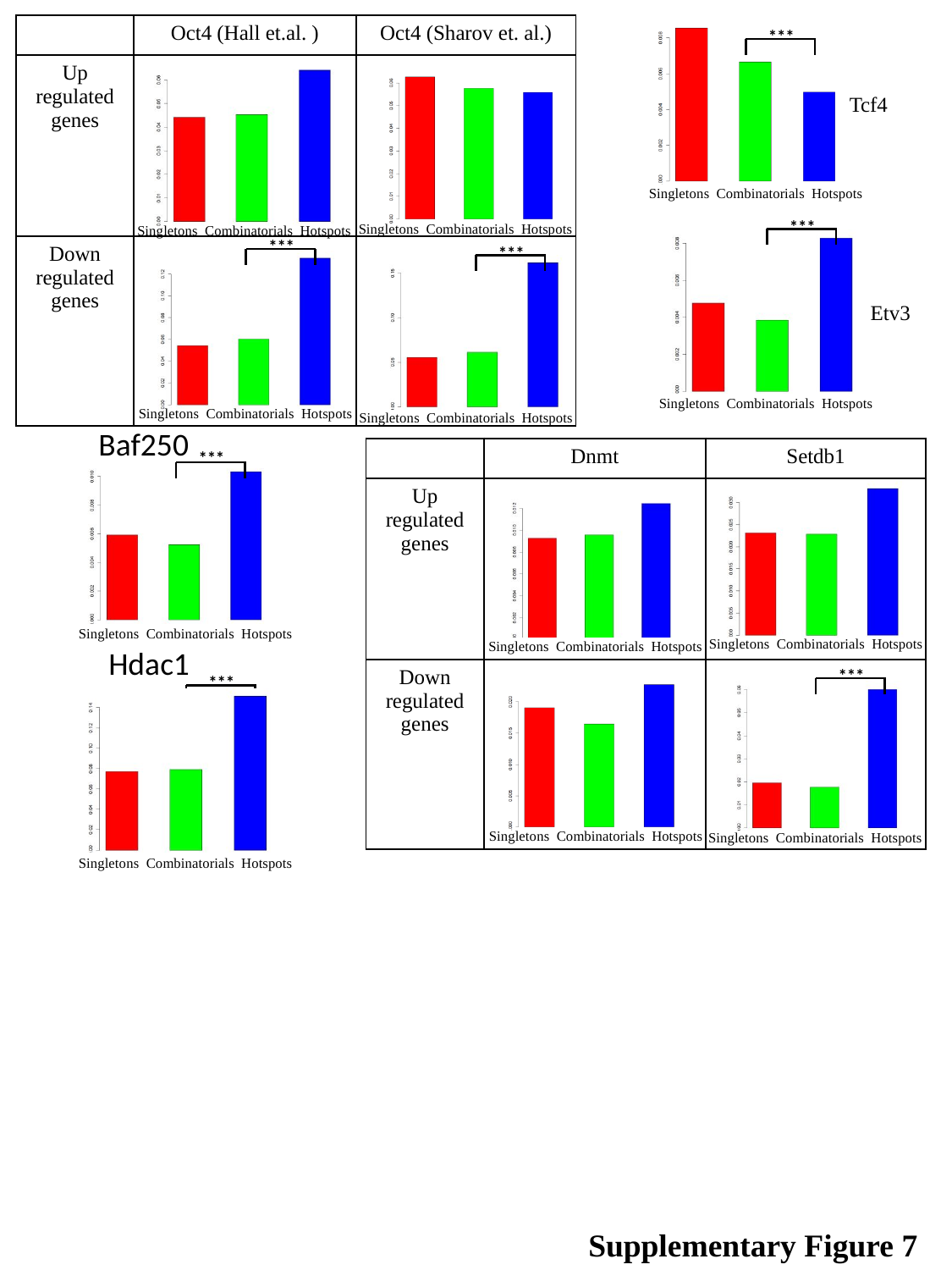

| | Oct4 (Hall et.al. ) | Oct4 (Sharov et. al.) |
| --- | --- | --- |
| Up regulated genes | | |
| Down regulated genes | | |
***
Tcf4
Singletons Combinatorials Hotspots
***
Singletons Combinatorials Hotspots
Singletons Combinatorials Hotspots
***
***
Etv3
Singletons Combinatorials Hotspots
Singletons Combinatorials Hotspots
Singletons Combinatorials Hotspots
Baf250
| | Dnmt | Setdb1 |
| --- | --- | --- |
| Up regulated genes | | |
| Down regulated genes | | |
***
Singletons Combinatorials Hotspots
Singletons Combinatorials Hotspots
Singletons Combinatorials Hotspots
Hdac1
***
***
Singletons Combinatorials Hotspots
Singletons Combinatorials Hotspots
Singletons Combinatorials Hotspots
Supplementary Figure 7
